# Compound specific isotope analysis of lipid residues provides the earliest direct evidence of dairy product processing in South Asia

**DOI:** 10.1038/s41598-020-72963-y

**Published:** 2020-09-30

**Authors:** Kalyan Sekhar Chakraborty, Greg F. Slater, Heather M.-L. Miller, Prabodh Shirvalkar, Yadubirsingh Rawat

**Affiliations:** 1 Department of Anthropology, University of Toronto, Mississauga, ON Canada; 2 School of Geography and Earth Sciences, McMaster University, Hamilton, ON Canada; 3 Department of A.I.H.C. and Archaeology, Deccan College Postgraduate and Research Institute, Pune, Maharashtra India; 4 State Department of Archaeology and Museum, Gandhinagar, Gujarat India

**Keywords:** Biogeochemistry, Environmental social sciences, Environmental chemistry, Organic chemistry

## Abstract

The early evidence of domesticated animals and human–animal interaction in South Asia can be traced back to the seventh millennium BCE; however, our understanding of their use is incomplete and limited to the analysis of animal bones from archaeological sites. By the third millennium BCE with the emergence of the Indus Civilization, cattle and water-buffalo became the primary domesticates and outnumbered any other animals at the majority of the Indus settlements. Based on the analysis of skeletal remains and ethnographic data, a number of studies have suggested that cattle and water-buffalo were utilized for their meat, dairy, hides, and other labor-oriented jobs. While some of these claims are backed by empirical data, others are primarily discussed as hypotheses, for example, the exploitation of dairy. In this paper, by analyzing the absorbed lipid residues from fifty-nine ceramic sherds recovered from an agro-pastoral settlement that was occupied during the peak of the Indus period around mid- to late third millennium BCE, we provide the earliest direct evidence of dairy product processing, particularly from cattle and possibly from some water-buffalo. By providing direct evidence of animal product processing, we identify the use of primary domesticated animals and other resources in the diet during the Indus Civilization.

## Introduction

**Figure 1:**
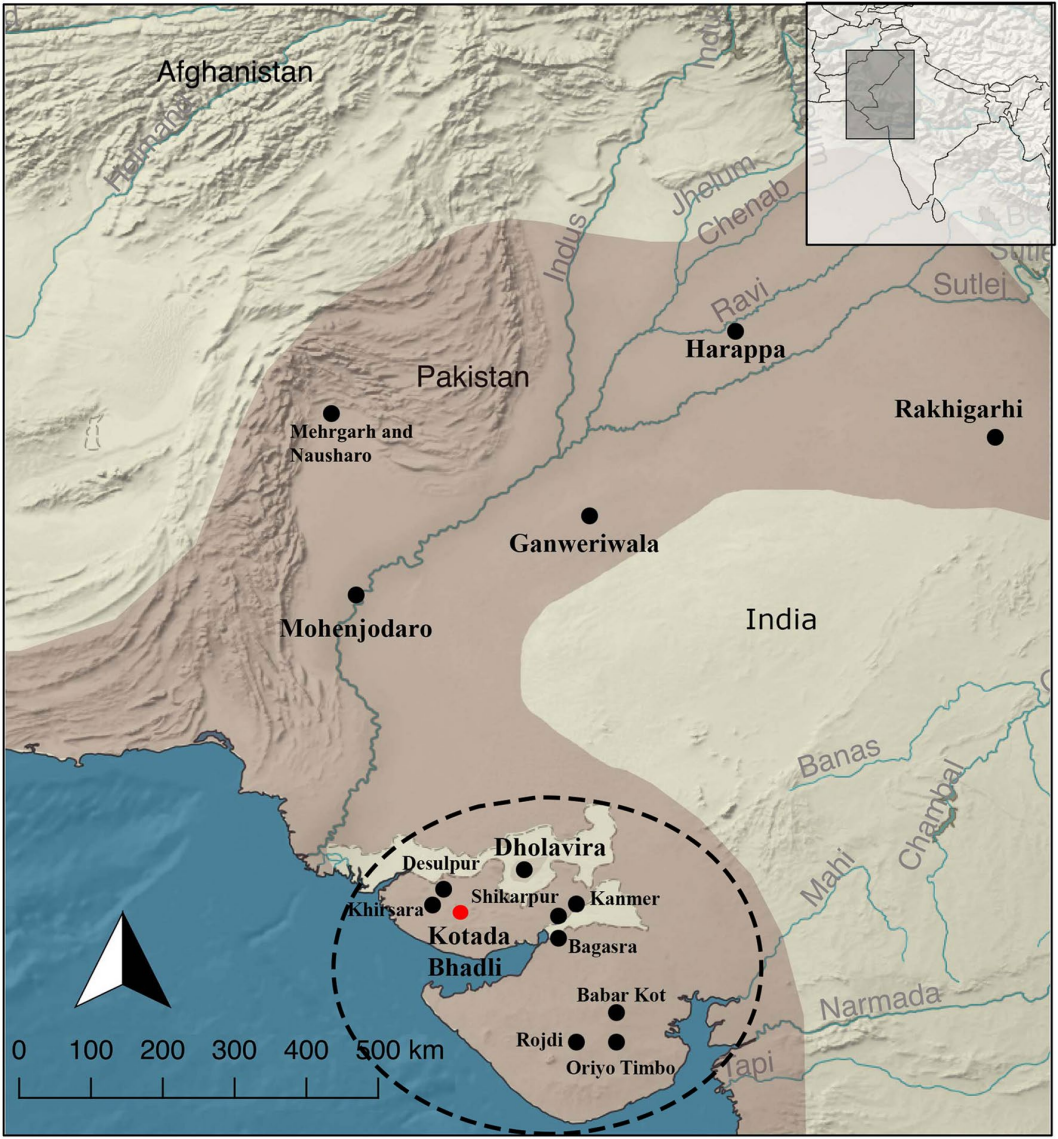
Highlighted map of the Indus Civilization showing the location of major settlements and settlements that are mentioned in this paper. The black dashed lines indicate the region under study and the red dot indicates the site under study. The map was created using QGIS Desktop version 2.18.14 (www.qgis.org), and raster and vector map data was acquired from Natural Earth (https://www.naturalearthdata.com/), which is available in public domain. The design idea with permission was adapted (Fig. 1, page no: 3)^17^.

The primary and secondary consumption of domesticated animals is a topic of great interest for archaeologists, particularly for those who are interested in early domestication^1–3^. It is now established that the earliest evidence of domestication in South Asia can be dated back to the seventh millennium BCE, based on evidence from the Aceramic Neolithic deposits at Mehrgarh. By the beginning of the fourth millennium BCE, domesticated animals such as cattle, water-buffalo, goat and sheep were present at most of the archaeological settlements throughout northwestern South Asia. During the onset of the third millennium BCE, economies depending on domesticated animals were prominent^4,5^, and by the mid-third millennium BCE archaeological data clearly demonstrates that all four of these animals were of major importance in the Indus Civilization (Fig. 1), and that specialized animal husbandry had become one of the primary economies^6^. Recent studies dealing with the interrelationships between humans, animals, and plants aim to understand the nature of plant and animal exploitation^5,7–13^, how people involved in these activities coped with changing environmental conditions^14,15^, and the socio-political dimensions of plant and animal exploitation^16–22^. Over a hundred years of excavations at many sites throughout the Indus Civilization region suggest that cattle, water-buffalo, and likely sheep were utilized for their meat and hides as well as for secondary products such as dairy, wool, and labor, while goats were primarily exploited for their meat and hides. While we have direct evidence of labor-oriented exploitation of cattle and water-buffalo in the form of traction-induced bone modification^23^, our knowledge of dairy exploitation during the Indus Civilization in particular, and South Asia in general, is limited. Here we will discuss the earliest evidence of secondary animal consumption in the form of dairy during the Indus period, the mid- to late third millennium BCE.

Having the ability to exploit animal products without the need to slaughter them may have enabled the acquisition of surpluses in secondary animal products that could be utilized for both regional and interregional exchanges, without affecting the size of a herd. Numerous suggestions of dairy consumption during the Indus Civilization have been made based on the mortality pattern of the animals^17,22,23^ as well as by considering artefactual remains that may represent probable consumption of dairy products^23^. However, none of these studies have provided any direct evidence of dairy consumption during the Indus period. The only lipid residue analysis of Indus/Harappan vessels prior to this research project was of a perforated vessel from Nausharo^24^. This study compared the fatty acids distribution of perforated vessels with the fatty acids of dairy from modern farm animals and suggested that these types of vessels were likely used for dairy product processing. The technique used in this study is now outdated and it does not provide conclusive evidence confirming the presence of dairy fat^25^.

The analysis of absorbed lipid residues in unglazed ceramic vessels is capable of providing direct evidence of dairy product processing^26–30^. Since the 1990s, researchers have frequently used the δ^13^C composition of the most abundant fatty acids C_16:0_ (palmitic acid) and C_18:0_ (stearic acid) to identify the source of lipids in archaeological pottery^26,31–38^. Beyond indicating the primary food sources, the offset in δ^13^C between C_16:0_ and C_18:0_ fatty acids has been used as a specific indicator for the presence of dairy lipids. This offset is calculated as Δ^13^C = δ^13^C C_18:0_–δ^13^C C_16:0,_ and it has been shown in multiple studies that a Δ^13^C value lower than − 3.1‰ would indicate ruminant dairy^38–40^. A plot of the δ^13^C values of C_18:0_ or C_16:0_ against the Δ^13^C values can be used to identify fats originating from animals that predominantly consumed C_3_ type vegetation vs. animals that predominantly consumed C_4_ type vegetation^41,42^. In recent years, the analysis of lipid residue from archaeological vessels has been successfully used to determine the processing of other products as well, such as aquatic product processing^43^, cheese-making^44^, processing of plants to produce alcoholic beverages^45^, and the type of oil used in lamps^46^. Lipid analysis has also helped in the identification of resinous materials that have been used as adhesive and waterproofing layers on ceramic vessels^47–50^. This tool has been successfully used to identify the sources of organic materials in wall paintings^51,52^ and in ashy deposits from archaeological settlements^53^.

### Archaeological data from the settlement under study

**Figure 2:**
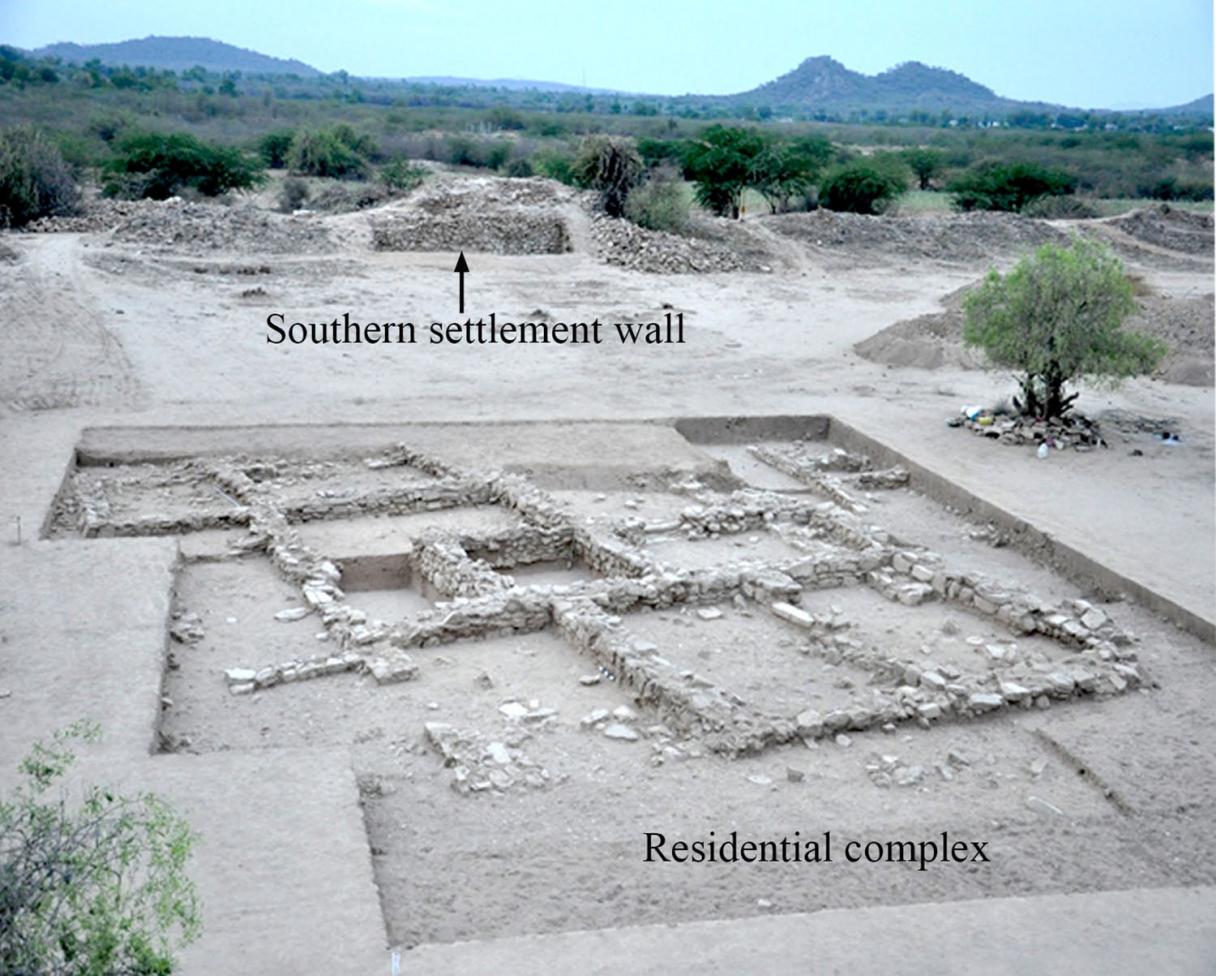
Excavated area of Kotada Bhadli, showing the southern settlement wall and the central residential complex with ten interconnected rooms. Picture was taken during the excavation by one of the authors.

For the present study, the settlement of Kotada Bhadli (23°20′N; 69°25′E), which is located in the Nakhatrana Taluka of District Kachchh, Gujarat was selected, as previous research by the primary author suggests that a sedentary to semi-sedentary form of animal husbandry was the primary occupation at this settlement^54,55^ (Fig. 1). This site measures around 3.11 ha and is surrounded by a settlement wall. The excavations from 2010 to 2013 have unearthed a central residential complex with ten interconnected rooms, and the recent AMS dates suggest that it was occupied between 2300 and 1950 BCE^55,56^ (Fig. 2). The preliminary zoo-archaeological report indicates that cattle, possibly some water-buffalo, goats and sheep were the primary domesticated animals consumed at this settlement, along with *Sus* species, although the identification of the *Sus* as domesticated is ambiguous^57^. A similar distribution of domesticated animals can be observed at other settlements in this region during the Indus period^13,17,58–60^; however, water-buffalo were possibly not a major domesticated animal in Kachchh^17,61^ (except perhaps at Dholavira^61,62^). The strontium isotope values from the tooth enamel of the primary domesticated animals at the site of Kotada Bhadli indicate that cattle, water-buffalo, goat, and sheep were possibly raised locally, and the carbon isotope values indicate that human-induced foddering played a major role in the rearing of these domesticates^54^.

### Animal rearing at Kotada Bhadli and its possible effect on lipid residue data

A complete zoo-archaeological study of animal remains at the settlement of Kotada Bhadli is underway at the time of writing. A preliminary study of twenty individual tooth samples suggests that most of the cattle and water-buffalo died at an older age, suggesting production of secondary products, whereas the majority of goat/sheep died when they were young, indicating their use primarily for meat; the sole sample identified as possibly sheep also died at an older age^54^. This preliminary study is based on a limited number of samples and may not be representative of the entire assemblage from the settlement; however, such a kill-off pattern is similar to those found at other contemporary settlements in the same region, where the majority of cattle/water-buffalo lived into adulthood and the majority of sheep/goats were slaughtered when young, with some kept alive to maintain herd numbers^16–18,22^. Such a pattern would not result from the exploitation of cattle/water-buffalo exclusively for dairy products. If this were the case, one would expect to see the majority of young male cattle and water-buffalo slaughtered at a young age, in order to maximize milk production by maintaining a herd consisting primarily of adult females^17,61,63,64^. Instead, it has been argued that these culling patterns observed in Gujarat indicate that a large number of male cattle (bulls and bullocks) and possibly some water-buffalo were kept alive for traction and for labor-oriented jobs as well as for selective exchange of animals between settlements during the Indus period^17,61^.

Analysis of stable carbon isotopic ratios (reported as δ^13^C_(enamel)_) of biogenic remains (tooth enamel) of herbivorous animals can be used to distinguish between diets based on plants that follow a C_4_ photosynthetic pathway or a C_3_ photosynthetic pathway^65,66^. Prior to the Industrial Revolution, δ^13^C_(enamel)_ values between − 11.5‰ and − 6.5 would indicate a C_3_ dominated diet, whereas, for animals consuming a C_4_ dominated diet, the δ^13^C_(enamel)_ will be around 2.5‰^66–68^. At the settlement of Kotada Bhadli, the δ^13^C_(enamel)_ values from twenty individuals suggest that cattle/water-buffalo and the only possible sheep varies from − 2.9‰ to 1.5‰ with a median value of 0‰, whereas for goat/sheep, it ranges from − 10.7‰ to − 2.3‰ with a median value of − 5.5‰^54^. The results for biogenic isotope data of tooth enamel of cattle, water-buffalo, goat, and the one animal idenfied as likely a sheep at the site of Kotada Bhadli indicate that primarily cattle/water-buffalo and the possible sheep consumed a varied degree of agricultural fodder that followed a C_4_ photosynthetic pathway, along with occasional intake of C_3_ vegetation; goats/sheep primarily consumed vegetation that followed a C_3_ photosynthetic pathway^54^. Similar foddering practices for cattle/water-buffalo and goat/sheep has been observed at the neighbouring settlements of Bagasra, Shikarpur and Jaidak^22,90^ that were also occupied during Indus period. Cattle/water-buffalo and goat/sheep are often conflated in zooarchaeological studies, and it is difficult to individually identify them in general based on fragmentary skeletal remains. While the detailed analysis of archaeozoological material of the site is not yet complete, the preliminary zoo-archaeological study at Kotada Bhadli and at other nearby settlements also grouped these animals as cattle/water-buffalo and goat/sheep^13,17,22,57,59^.

Similarly to what is observed for tooth enamel, the δ^13^C values of C_16:0_ and C_18:0_ fatty acids from lipids would reflect the δ^13^C composition of the primary food sources of the animal, as the δ^13^C values of fatty acids in herbivorous animals reflects the δ^13^C values of both carbohydrates and fatty acids of plants that they consume. Many studies have used observed ranges of the δ^13^C values of C_16:0_ and C_18:0_ fatty acids to differentiate food sources based on C_3_ and C_4_ directly^37,39–42^. Due to such differences in the carbon isotope values between C_4_ and C_3_ plants^69^, animals primarily consuming C_4_ type vegetation would produce enriched δ^13^C values of C_16:0_ and C_18:0_ fatty acids for both dairy and adipose fats, compared to animals predominantly consuming C_3_ type vegetation^42^. Thus, measuring the δ^13^C values of C_16:0_ and C_18:0_ fatty acids can indicate the relative consumption of C_3_ and C_4_ vegetation. If cattle and/or water-buffalo were used for dairy exploitation, and if dairy was a major dietary constituent during the Indus period, we should be able to identify it based on the compound specific isotope analysis of the most abundant fatty acids.

## Material and method

### Samples

**Figure 3:**
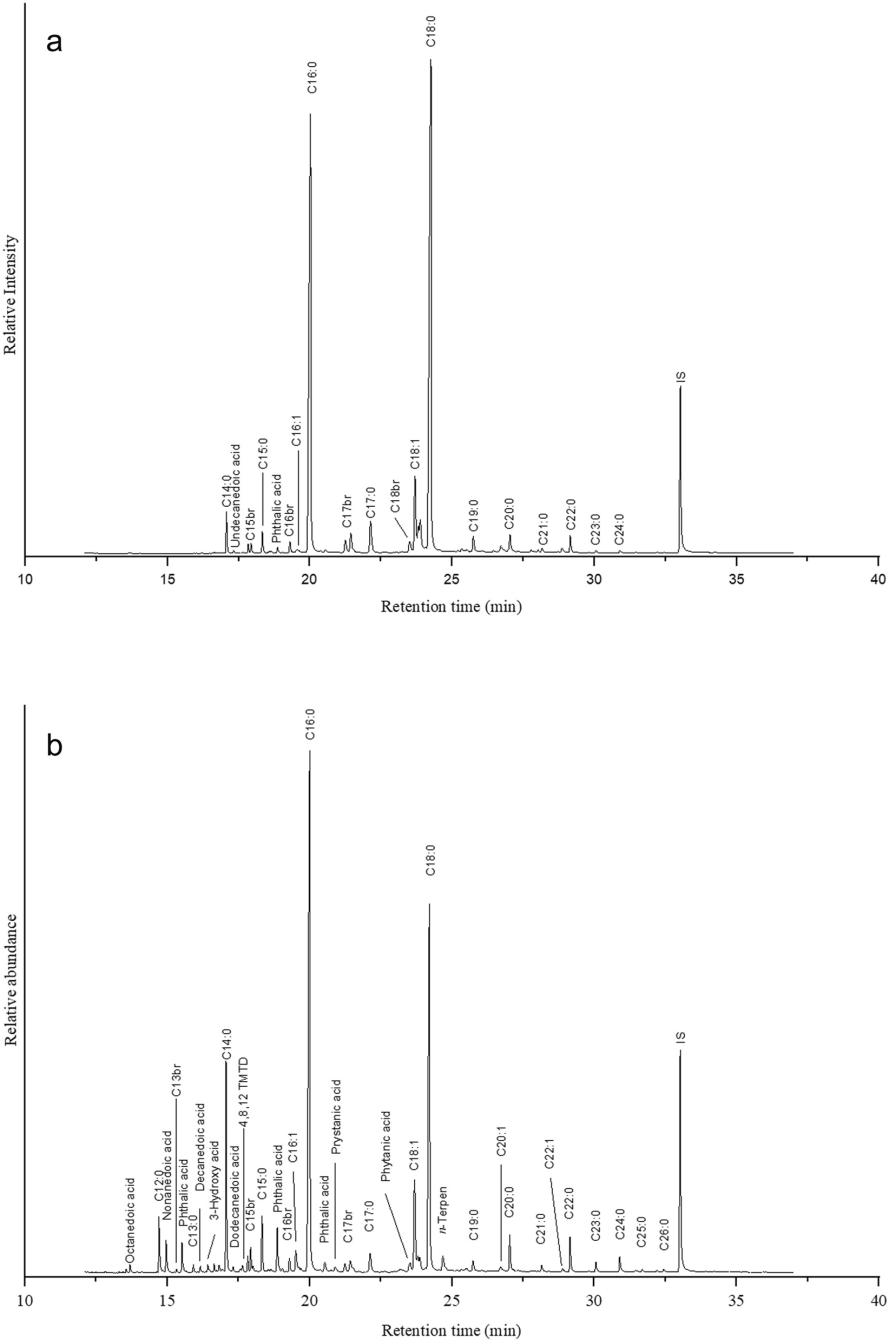
Partial gas chromatograms of TLEs from two Sorath Harappan potsherds from the site of Kotada Bhadli. Peaks were identified by GC–MS. Numbers (*X:Y*) refer to chain lengths (*X*) and number of saturation (*Y*) in the methanolic H_2_SO_4_ extracts of the fatty acids; br indicates the presence of branched chain fatty acids and IS is the internal standard. (**a**) is the partial gas chromatogram of KB-171 indicating degraded animal fat and (**b**) is the partial gas chromatogram of KB-106 indicating degraded possible aquatic fat.

Fifty-nine uncleaned pottery fragments from throughout the settlement of Kotada Bhadli were collected from both the residential area and near the settlement wall during the excavations of 2011–2012 and 2012–2013 (see Supplementary Information table S1 and Fig. 2). The area near the wall was used to dump occupational debris during the site’s occupation. The majority of sherds collected were from cooking vessels and various type of bowls; fragments of a perforated jar, pots, ladles, lids and unidentified coarse red-ware vessels were also included in this sample (Fig. 3, Supplementary Information table S1 and S2).

Upon excavation, these pottery fragments were immediately transferred into paper bags without washing and were stored for analysis. Once in the lab, any sediment and/or calcite depositions on the pottery surfaces were removed using an abrader drill bit. No water was used in the process of cleaning; prior to sampling, any dust adhering to the surface of the sherds was removed using pressurized air flow. Approximately 5 gm of cleaned ceramic was powdered using an agate mortar, and stored in aluminum foil that was previously baked at 450 °C. All the tools used for cleaning and powdering were cleaned after each use, using RO water and organic solvents.

### Extraction

One-step acidified methanolic extraction^70^ was used as the primary method to extract absorbed organic residues from all the 59 samples. Along with this, 5 samples were analyzed using conventional chloroform–methanol extraction and BSTFA derivatization^71^ (see Supplementary Information for the detailed methodology) in order to identify the possible presence of compound lipids and any underivatized hydroxyl groups that the rapid and more aggressive acidified method may have failed to identify^70^. These samples were chosen based on the fact that they had extractable fatty acids both higher and lower than the settlement mean, and in the acid extracts of those samples, the distribution of fatty acids and the ratio between C_16:0_ and C_18:0_ fatty acids indicated the presence of plant oil, animal fat and aquatic fats. Results published elsewhere^55^ suggest no preservation of any complex lipids such as glycerids, sterols, waxes and alcohols in the conventional extract; the rapid and efficient one-step acidified methanol extraction^29,70,72–75^ was therefore chosen over the conventional method because it produced high lipid residue yields from the very old and poorly preserved ceramic sherds of Kotada Bhadli^55^. Briefly, for each sample, 2 gm of ceramic powder was weighed and transferred into a 40 ml glass vial (vial 1), and 5 ml of MeOH–H_2_SO_4_ (2% *v/v*.) was added and left for one hour at 70 °C, while shaken every 10 min to extract fatty acids from ground ceramic powders. The pH of the extraction was monitored to maintain a pH level < 3. After the extraction, the supernatant was transferred into another 10 ml vial (vial 2), and 2 ml of DCM extracted double distilled water was added, then 3 ml of hexane was added to vial 2, vortexed, and the hexane layer with Fatty Acid Methyl Ester (FAME) was transferred to another 10 ml vial (vial 3). Following this, 3 ml of hexane was added to vial 1 to extract any lipids not fully solubilized by the methanol solution. The hexane layer from vial 1 was then poured into vial 2 and whirl mixed, and then was finally transferred to vial 3. This step was repeated thrice, and all the hexane layers were mixed together in vial 3; finally, it was taken to a virtual dryness under a gentle stream of nitrogen. It was re-dissolved and following a standard protocol^73,75,76^, prior to the GC-MS and GC-IRMS analysis 20 μl of *n-*triacontane (1 mg/ml) was added as an internal standard for quantification purposes, and then the contents of vial 3 were transferred into a 300 μl insert for GC–MS and GC-IRMS analysis. As our aim was to get high yields of fatty acids especially for compound specific isotope analysis, we did not analyze all of our samples using conventional extraction, neither did we derivatize our acid extracts to recover high polar compounds. Due to the limitation of direct transesterification, however, any study wishing to extract high polar compounds, such as alcohols, require that an aliquot must be derivatized with BSTFA prior to GC–MS analysis.

### GC–MS analysis

The analysis was carried out at McMaster University, Ontario, Canada, on an Agilent 6890 GC equipped with a 5973 quadrupole mass spectrometer. The column used was an Agilent DB5-MS + DG, 30 m × 0.25 mm with a 0.25 μm thickness. To achieve a better separation of peaks, the initial GC temperature was set at 50 °C and held for two minutes, ramped to 200 °C at a rate of 10 °C/min and held for ten minutes, again ramped to 300 °C at a rate of 10 °C/min and held for 10 min. 1 μl of sample was introduced to the GC by splitless injection. The MS was operated in a scan mode with 12 min of sample delay, the MS quad temperature was set at 150 °C and the MS source temperature was set at 230 °C. The data acquisition was between *m/z* 50 and 450. Acquisition and data analysis were performed using ChemStation D.01.02 software.

### GC-IRMS analysis

The GC-IRMS analysis was also carried out at McMaster University, Canada. Based on the concentration of C_16:0_ and C_18:0_ fatty acid methyl esters, 21 ceramic sherds were selected for the GC-IRMS analysis. As a precautionary cleaning step prior to GC-IRMS analysis, a secondary liquid chromatography separation of FAMES was carried out using silica gels and a number of organic solvents of increasing polarity^37,77^, and the fractions containing hydroxy fatty acids were selected for the GC-IRMS analysis. Samples were either concentrated or diluted for GC-IRMS analysis. The gas chromatographic analysis was performed on an Agilent 6890 GC coupled with a Thermo-Finnigan DeltaPlus XP isotope ratio mass spectrometer via a Conflo-III interface. To achieve a better separation between unsaturates and saturates, the sample separation was performed on an HP-88, 100 m × 0.25 mm with a 0.20 μm thickness column. Injection was achieved through a splitless injector at 310 °C with an injection volume of 2 μl. The column was connected to a 1 m deactivated pre-column. Helium was the carrier gas. The temperature program was 80 °C held for 1 min, ramped to 175 °C at 10 °C/min and held for 12 min, ramped to 190 °C at 2 °C/min with a 10 min hold, finally ramped to 240 °C at 10 °C/m with a hold for 15 min. Acquisition and data analysis were performed using Isodat 2.03 software. Analytical accuracy was confirmed via isotopically characterized standards run before and after each set of samples. Accuracy and precision on triplicate sample analysis was between 0.1 and 0.7 per mil (2 sigma). The isotope values of the samples have been corrected by the known δ^13^C value of the methanol used for the extraction. The mathematical formula below has been used for the correction:

$$\delta^{{{13}}} {\text{C}}_{{{\text{FA}}}} \, = \,\left[ {\left( {{\text{C}}_{{\text{n}}} \, + \,{1}} \right)\, \times \,\delta^{{{13}}} {\text{C}}_{{{\text{FAME}}}} - \delta^{{{13}}} {\text{C}}_{{{\text{MeOH}}}} } \right]/{\text{C}}_{{\text{n}}}$$

Here C_FA_ is the corrected δ^13^C value of the fatty acids, C_n_ represents the number of carbon atoms in the chain, δ^13^C_FAME_ is the observed carbon isotope value of the fatty acids and δ^13^C_MeOH_ is the known carbon isotope value of the methanol used for the extraction of fatty acids.

## Results and discussion

**Figure 4:**
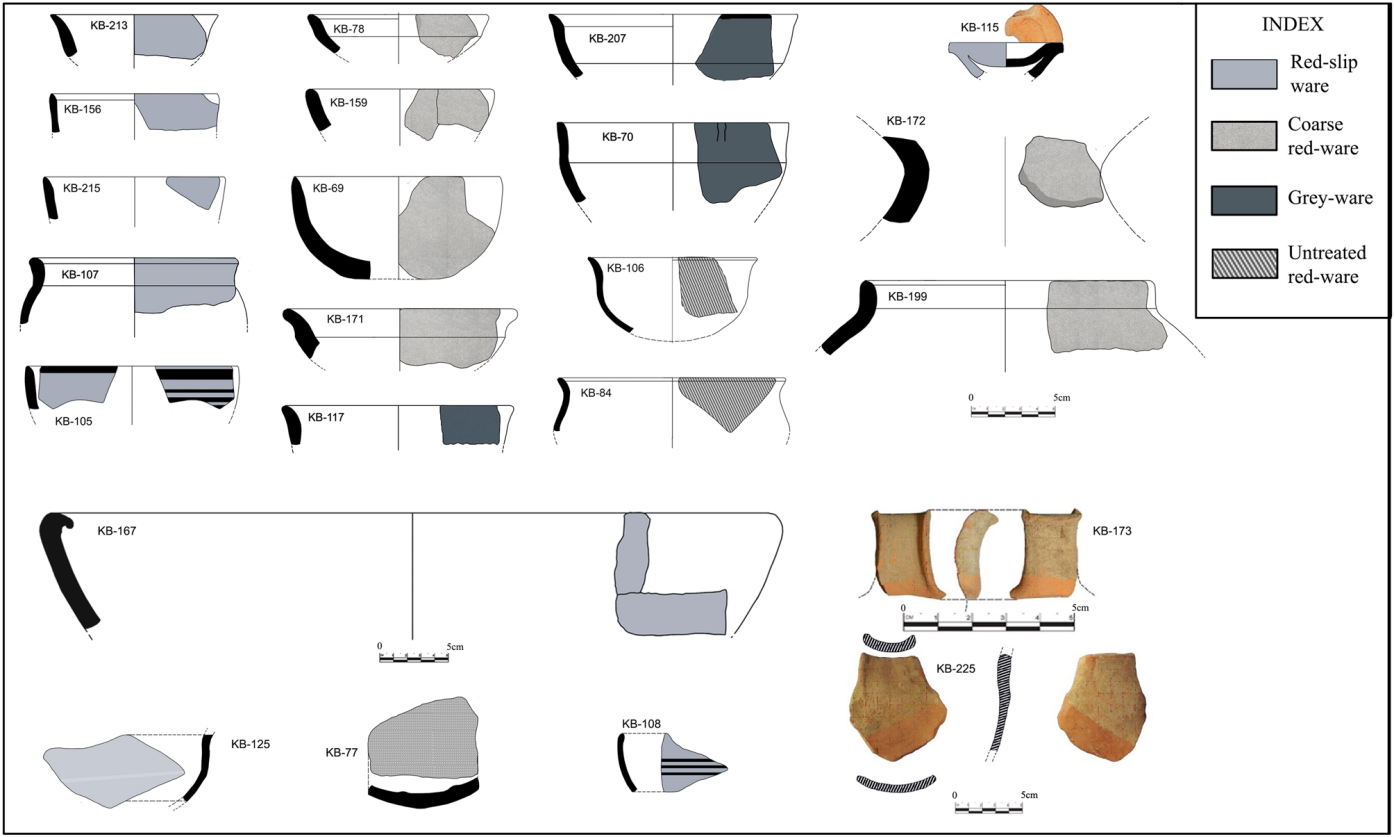
Reconstructed vessels from the settlement of Kotada Bhadli used for organic residue analysis.

The amount of lipid residues extracted from Kotada Bhadli pottery fragments ranged between 5–262 μg/g (mean = 25 μg/g). The distribution of fatty acids in the samples from Kotada Bhadli (SI table 1) were dominated by medium to long chain saturated fatty acids (C_12:0_ to C_20:0_), unsaturated fatty acids (C_16:1_ and C_18:1_), and branched chain fatty acids (C_15br_ to C_18br_). In rare occasions, very long chain saturated fatty acids (C_21:0_–C_28:0_), and long chain unsaturated fatty acids (C_18:2_, C_20:1_ and C_22:1_) were identified. The other molecules that were present in many of these samples include dicarboxylic acid (C_8_–C_11_), isoprenoid acids (phytanic acid, pristanic acid, 4,8,12 trimethyltridecanoic acid), and aromatic hydrocarbons. The contaminants included phthalic acids (see Fig. 4 and Supplementary Information table S1).

This research has been designed to identify the earliest usage of dairy from ruminant animals as well as the utilization of adipose fats from ruminant and monogastric animals in the diet during the Indus period. Therefore, twenty-one samples out of 59 samples were selected for GC-IRMS analysis, as they had indications of the preservation of animal fats. Following the most common practice in archaeology^40,70,72–74^, the samples with higher concentrations of C_16:0_ and C_18:0_ fatty acids, those indicative of animal fats, were selected. Along with the high abundance of C_16:0_ and C_18:0_ fatty acids, the presence of odd chain fatty acids (C_13:0_ to C_19:0_) and branched chain fatty acids (C_15:0br_ to C_18:0br_) were used to tentatively identify ruminant fats^40,70^ (see Supplementary Information table S1). A low abundance of C_18:0_ fatty acid as compared to C_16:0_ fatty acid, which is often associated with the presence of plant oils^40,78^, were observed in six samples (Supplementary Information table S1). These six samples were not included in the compound specific isotopic analysis as we focus on animal utilization at the settlement of Kotada Bhadli.

One out of the 59 samples, a red-ware untreated bowl, contained some evidence of aquatic fat (this sample was excluded from the isotopic analysis). This tentative identification was based on the presence of isoprenoid acids (phytanic acid, pristanic acid, 4,8,12 TMTD), a low concentration of ω-(*o-*alkylphenyl) alkanoic acids, and long chain mono-unsaturated fatty acids particularly C_20:1_ and C_22:1_, that are indicative of the presence of aquatic fats^29,34,79,80^. A selected ion search (*m/z* 105, 290, 318 and 346) suggested a possible presence of ω-(*o-*alkylphenyl)alkanoic acids 18, 20 and 22 carbons in length that are formed by heating of polyunsaturated fatty acids generally present in aquatic organisms^34,80^. No other samples from Kotada Bhadli indicate similar distributions of fatty acids indicative of aquatic fats. Due to the distance of Kotada Bhadli from the sea, it is unlikely that marine fats played a major role in the diet of the residents. While we cannot completely rule out the possibility of occasional consumption of aquatic fats from the nearby seasonal rivers, the zoo-archaeological studies at this site^57^ and at its neighbouring sites^13,16,58,59,61^ suggest that although aquatic fats were consumed, they were never a major constituent of Harappan diet in this region.

**Figure 5:**
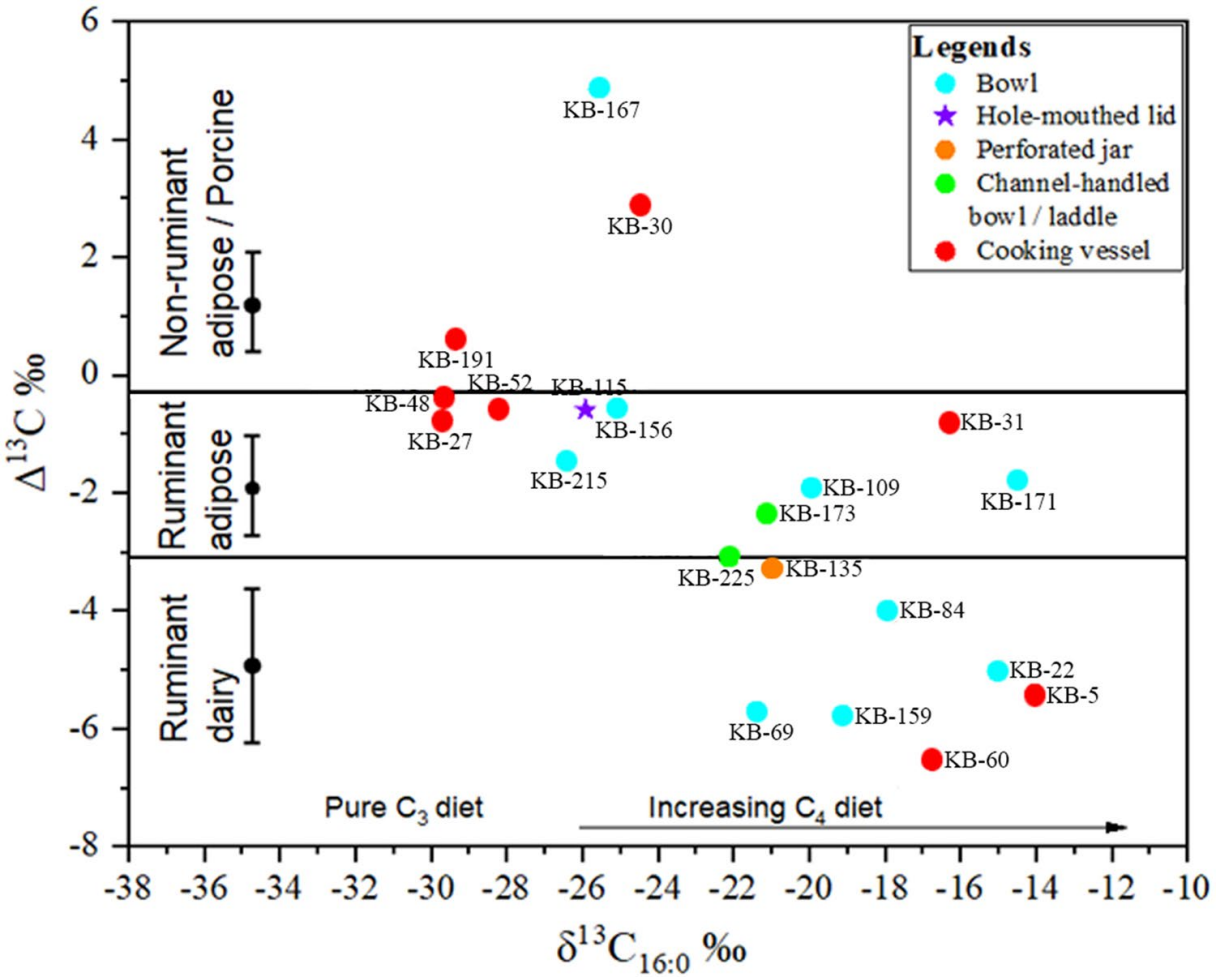
Plots of Δ^13^C values from Kotada Bhadli pot sherds placed against the δ^13^C values of C_16:0_ fatty acids of the same potsherds. The ranges shown here represent the mean ± 1 s.d. of the Δ^13^C values for a global database comprising modern reference animal fats from Africa, UK, Kazakhstan, Switzerland, and the Near East^39^.

The observed range of δ^13^C values of C_16:0_ and C_18:0_ fatty acids of the 21 samples are from − 14‰ to − 29.7‰, and from − 16.3‰ to − 30.5‰ (Supplementary Information table S2 and Fig. 1). Interestingly, a wide range in the δ^13^C values of C_16:0_ and C_18:0_ fatty acids and distinct groupings (Fig. 5) were observed in the Kotada Bhadli samples. The biogenic carbon isotope data of tooth enamel from major domesticated animals^54,55^ earlier suggested that due to selective foddering practices at Kotada Bhadli, one group of domesticated animals (cattle, water-buffalo and one likely sheep) ate a considerable portion of C_4_ type vegetation, whereas, the other group of domesticated animals (indistinguishable goats/sheep) ate primarily C_3_ vegetation. As we hypothesized earlier, this foddering practice has influenced the variation in the observed range of δ^13^C values of C_16:0_ and C_18:0_ fatty acids. The distinct groupings (Fig. 5) suggest that ruminant animal fats from the nine samples that produced δ^13^C (C_16:0_) values between − 24‰ and − 30‰ were influenced by a C_3_ rich diet, and in case of Kotada Bhadli, were possibly goat/sheep or other ruminant animals that predominantly consumed C_3_ type vegetation. Ruminant fats from the remaining 12 samples that produced δ^13^C (C_16:0_) values between − 22‰ and − 14‰ indicate the influence of C_4_ in their diet, which were likely to be coming from cattle, some water-buffalo, and possibly from some sheep (based on the enamel isotope data from a single likely sheep from Kotada Bhadli^54^), which is also expected because cattle and possibly some water-buffalo were the primary domesticated animals at the settlement of Kotada Bhadli^57^.

The Δ^13^C_(C18:0-C16:0)_ values of the 21 samples selected for isotopic analysis varied between 4.9‰ and − 6.5‰, indicating the presence of both ruminant and non-ruminant adipose fat as well as ruminant dairy fat (Fig. 5). When Δ^13^C values are plotted against the δ^13^C (C_16:0_) values (Fig. 5), it appears that all the ruminant dairy fats in our assemblage were exploited from cattle, some water-buffalo, and possibly from some sheep that consumed primarily C_4_ type vegetation; adipose fats were coming from both sheep/goat and cattle/water-buffalo, as well as from monogastric animals, likely pig. Unfortunately, the modern foddering pattern in this region does not replicate the foddering pattern that we observed at the site of Kotada Bhadli^54,55^, limiting our ability to develop a site-specific modern reference collection. At present, C_3_ vegetation and cash crops play a major role in animal foddering as compared to C_4_ type vegetation, such as millets.

As suggested above, dairy residue from any ruminant animal that consumed predominantly C_4_ type vegetation at this settlement may produce similar enriched δ^13^C(C_16:0_) values, whether cattle, water-buffalo, or possibly sheep. However, sheep were never a primary domesticated animal at this settlement^57^ or at the neighbouring settlements^13, 17,22,59,61^, and those found in the eastern Indus regions are proposed as exploited for meat and wool^81^. Cattle and water-buffalo (depending on the region) were the primary domesticated animals, ranging from 60–90% of the assemblage during the Indus period, not only in Gujarat, but also in the other parts of the Indus Civilization^5,13,17,22,57,59,61^. It is therefore reasonable to argue that dairy, which may have been an integral part of everyday diet at Kotada Bhadli, was likely to be exploited primarily from cattle, and possibly from some water-buffalo, although occasional exploitation of dairy from sheep and goats is also a possibility. Due to the expansion of the Indus Civilization into vastly different geological and environmental zones and the possibility of local adaptations, our observations from Kotada Bhadli likely represent one of several Indus animal-exploitation strategies.

By taking a slightly conservative approach and allowing for the possibility of mixing between dairy and adipose fats, as well as probable occasional consumption of deer meat, as suggested by the presence of uncommon deer skeletal remains at this settlement and from the neighboring sites during the Indus period^13,57,59^, the Δ^13^C adipose fat of ruminant animals can be extended up to − 4.3‰^82^. Even with this adjustment, it is clear that there are at least five samples that produced negative Δ^13^C below − 4.3‰ and can be securely identified as dairy fat at Kotada Bhadli. It is important, however, to keep in mind that deer have never been considered to be a major constituent of Indus/Harappan diet and were likely to be consumed only occasionally. In addition, all samples indicating the presence of dairy fats have originated from animals that predominantly consumed varying degrees of C_4_ type vegetation. In the case of Kotada Bhadli these would primarily be agricultural millets^54,55^, and it is highly unlikely that wild ruminant animals of that region consumed such a high proportion of agricultural vegetation.

Cooking techniques and multiple usage of serving vessels may have resulted in some mixing between animal fats and plant oils; little work has been done on cooking techniques, but cut marks on the bones from this region suggest that both stewing and roasting may have been a standard way of processing meat during the Indus period at least in Gujarat^16^. Mixing between animal fats can be observed in our samples (see Supplementary Information table S2); however, it is difficult to ascertain the mixing between animal fats and plant oils. Oil seeds used in this region all fall under the C_3_ category of plants^9,13,15,83^; they are unlikely to produce δ^13^C values of fatty acids similar to the values influenced by the C_4_ photosynthetic pathway, which we have observed for all of our dairy and in some of the adipose fats primarily exploited from cattle/water-buffalo. Also, as previously observed^84,85^, the Δ^13^C_(C18:0–C16:0)_ values of plant oils are generally indistinguishable from ruminant and non-ruminant adipose fats, but they are not similar to the values of dairy fats. On the other hand, cereal grains have low lipid content which can be easily overshadowed by animal fats^73,86,87^. Recent studies have pointed out that mixing between animal fats and plant oils can potentially affect the Δ^13^C values, based on simulated results^88,89^. While mixing of ruminant adipose fats with C_3_ plant oils can never produce Δ^13^C values similar to dairy, the simulated mixing of C_3_ ruminant fats with C_4_ plants was shown to be able to produce dairy Δ^13^C values^89^. This is particularly crucial in this present scenario when various millets that are primarily C_4_ in nature were possibly consumed by both human and cattle/water-buffalo in Gujarat, whereas goats/sheep were primarily raised on C_3_ type vegetation^22,54,90^.

There are several lines of evidence that support that mixing of plant oils and animal fats were not responsible for the observed offsets in the Δ^13^C_(C18:0–C16:0)_ values of dairy samples at the settlement of Kotada Bhadli. First, a thorough search for miliacin, a biomarker of broomcorn millet, did not reveal its presence in any of these samples. Further, the samples analyzed were selected such that the ratio between C_16:0_ and C_18:0_ and the distribution of fatty acids in samples chosen for isotopic analysis were consistent with animal fats rather than plant sources. But perhaps most importantly, due to the fact that plant oils generally contain high levels of C_16:0_ and low levels of C_18:0_ fatty acids, addition of plant oils would be expected to result in a shift of C_16:0_ values but not in C_18:0_ values resulting in a Δ^13^C_(C18:0–C16:0)_ offset consistent with dairy. Noticeably both the C_16:0_ and C_18:0_ fatty acids of the sample indicating dairy consumption were enriched because of the C_4_ dependent selective foddering of cattle/water-buffalo at this settlement (Supplementary Information table S2 and Fig S1). In addition, because mammary glands in ruminant animals are unable to biosynthesize C_18:0_, whereas, a major portion of it in the adipose fats are due to de nevo biosynthesis from acetate, it is therefore likely that the δ^13^C values of C_18:0_ fatty acids in adipose fats will be slightly enriched compared to the dairy fats of animals raised on the same diet^38^. This is what we observed for our samples, where the δ^13^C values of C_18:0_ in cattle/water-buffalo adipose fats were enriched compared to cattle/water-buffalo dairy fats (Supplementary Information table S2 and fig S1).

Based on these lines of evidence we feel that the potential mixing of C_4_ plant derived FAMEs with animal derived lipids raised on C_3_ type vegetation is unlikely to have occurred in these samples that we have securely identified as potential dairy fats.

## Conclusions

In this paper we have presented the earliest direct evidence of dairy product processing in South Asia. This evidence was derived from the absorbed lipid residues preserved in the ceramic assemblage recovered from Kotada Bhadli, a sedentary to semi-sedentary agro-pastoral settlement occupied from 2300–1950 BCE^55^, during the Mature/Urban Harappan period, also known as the Integration Era of the Indus Civilization. Our findings suggest that dairy was an integral part of the everyday diet of its residents; this dairy was explicitly acquired from cattle and possibly water-buffalo. Studies based on the analysis of artefacts^91^, bone morphology^23^ and mortality profiles^17,61^ have previously suggested that during the Indus period both cattle and water-buffalo were used extensively for traction as well as for their meat, milk, and hide. These large-bodied animals were viewed not just as commodities, but may also have played an important role in Indus ideology, as indicated by their depiction in seals, sealing/tokens, pottery, and terracotta figurines^92^. The antiquity of cattle and water-buffalo for dairy exploitation may date back to as early as the sixth millennium BCE, when they were first domesticated in South Asia^4^, necessitating further analysis of older materials.

The presence of dairy fat at the settlement of Kotada Bhadli was identified based on Δ^13^C plotted against δ^13^C (C_16:0_) values which indicates that they were exploited from animals raised on C_4_ type vegetation, and at Kotada Bhadli these were cattle/water-buffalo. Data from three other neighbouring settlements where analysis have been done also indicate that during the Indus period, cattle/water-buffalo were raised on C_4_ type vegetation^22,54,90^; however, the nature of their utilization at these neighbouring settlements remains unidentified. Along with the earliest evidence of dairy use from South Asia, in this paper we have also provided supplementary evidence to support the idea that adipose fats from cattle/water-buffalo, goat/sheep, and monogastric animals were processed for consumption at this site, in line with zoo-archaeological work by Chase at nearby sites^16,17,93^. Additional work in the region is likely to provide significant findings about the versatility of Indus diet and the role of these usually invisible products in regional and international trade networks that developed during the Indus Civilization and played a crucial role in its survival and expansion.

## Supplementary information

Supplementary file1

Supplementary file2

## References

[1] Greenfield HJ (2010). The secondary products revolution: The past, the present and the future. World Archaeol..

[2] Marciniak A (2011). The secondary products revolution: Empirical evidence and its current zooarchaeological critique. J. World Prehist..

[3] Sherratt A (1983). The secondary exploitation of animals in the Old World. World Archaeol..

[4] Patel AK, Meadow RH, Albarella U, Rizzetto M, Russ H, Vickers K, Viner-Daniels S (2017). South Asian contribution to animal domestication and pastoralism: Bones, genes and archaeology. The Oxford Handbook of Zooarchaeology.

[5] Meadow RH, Patel AK, Weber SA, Belcher WR (2003). Prehistoric pastoralism in northwestern South Asia from the Neolithic through the Harappan period. Indus Ethnobiology: New perspective from the field.

[6] Wright RP (2010). The Ancient Indus: Urbanism, Economy, and Society.

[7] Fuller DQ, Weber S, Belcher WR (2003). Indus and non-Indus agricultural traditions: Local developments and crop adoptions on the Indian peninsula. Indus Ethnobiology.

[8] Meadow RH (1998). Pre- and Proto-Historic agriculture and pastoral transformations in northwestern and South Asia. Rev. Archaeol..

[9] Pokharia AK (2011). Archaeobotany and archaeology at Kanmer, a Harappan site in Kachchh, Gujarat: Evidence for adaptation in response to climatic variability. Curr. Sci..

[10] Rissman, P. C. Migratory Pastoralism in Western India in the Second Millennium B.C.: The Evidence from Oriyo Timbo (Chiroda). (University of Microfilms International, 1985).

[11] Weber S, Kashyap A, Harriman D (2010). Does size matter: The role and significance of cereal grains in the Indus Civilization. Archaeol. Anthropol. Sci..

[12] Weber SA (1991). Plants and Harappan subsistence: An example of stability and change from Rojdi.

[13] Goyal P (2013). Subsistence system, paleoecology, and 14C chronology at Kanmer, a Harappan site in Gujarat India. Radiocarbon.

[14] Pokharia AK, Kharakwal JS, Srivastava A (2014). Archaeobotanical evidence of millets in the Indian subcontinent with some observations on their role in the Indus Civilization. J. Archaeol. Sci..

[15] Pokharia AK (2017). Altered cropping pattern and cultural continuation with declined prosperity following abrupt and extreme arid event at ~4,200 yrs BP: Evidence from an Indus archaeological site Khirsara, Gujarat, western India. PLoS ONE.

[16] Chase B (2010). Social change at the Harappan settlement of Gola Dhoro: A reading from animal bones. Antiquity.

[17] Chase B (2014). On the pastoral economies of Harappan Gujarat: Faunal analyses at Shikarpur in context. Herit. J. Multidiscip. Stud. Archaeol..

[18] Chase B, Ajithprasad P, Rajesh SV, Patel A, Sharma B (2014). Materializing Harappan identities: Unity and diversity in the borderlands of the Indus Civilization. J. Anthropol. Archaeol..

[19] Belcher WR (2005). Marine exploitation in the Third Millennium BC- The eastern coast of Pakistan. Paleorient.

[20] Belcher WR (2003). Fish exploitation of the Indus Valley Tradition. Indus Ethnobiology.

[21] Chase B, Meiggs D, Ajithprasad P, Slater PA (2018). What is left behind: Advancing interpretation of pastoral land-use in Harappan Gujarat using herbivore dung to examine bioshphere strontium isotope (87Sr/86Sr) variation. J. Archaeol. Sci..

[22] Chase B, Meiggs D, Ajithprasad P, Slater PA (2014). Pastoral land-use of the Indus Civilization in Gujarat: Faunal analyses and biogenic isotopes at Bagasra. J. Archaeol. Sci..

[23] Miller LJ, Weber SA, Belcher WR (2003). Secondary products and urbanism in South Asia: The evidence for traction at Harappa. Indus Ethnobiology: New perspective from the field.

[24] Bourgeois G, Gouin P (1995). Résultats D ’ une analyse de traces organiques fossiles dans une ‘Faisselle’ Harappéenne. Paléorient.

[25] Evershed RP, Dudd SN, Copley MS, Mukherjee A (2002). Identification of animal fats via compound specific δ13C values of individual fatty acids: Assessments of results for reference fats and lipid extracts of archaeological pottery vessels. Doc. Praehist..

[26] Copley MS, Berstan R, Straker V, Payne S, Evershed RP (2005). Dairying in antiquity. II. Evidence from absorbed lipid residues dating to the British Bronze Age. J. Archaeol. Sci..

[27] Spangenberg JE, Jacomet S, Schibler J (2006). Chemical analyses of organic residues in archaeological pottery from Arbon Bleiche 3, Switzerland—evidence for dairying in the late Neolithic. J. Archaeol. Sci..

[28] Craig OE (2010). Stable isotope analysis of Late Upper Palaeolithic human and faunal remains from Grotta del Romito (Cosenza) Italy. J. Archaeol. Sci..

[29] Craig OE (2013). Earliest evidence for the use of pottery. Nature.

[30] Craig OE, Taylor G, Mulville J, Collins MJ, Parker PM (2005). The identification of prehistoric dairying activities in the Western Isles of Scotland: an integrated biomolecular approach. J. Archaeol. Sci..

[31] Evershed RP (2008). Organic residue analysis in archaeology: The archaeological biomarker revolution. Archaeometry.

[32] Craig OE, Love GD, Isaksson S, Taylor G, Snape CE (2004). Stable carbon isotopic characterisation of free and bound lipid constituents of archaeological ceramic vessels released by solvent extraction, alkaline hydrolysis and catalytic hydropyrolysis. J. Anal. Appl. Pyrolysis.

[33] Dudd SN, Evershed RP (1998). Direct demonstration of milk as an element of archaeological economies. Science.

[34] Buonasera TY, Tremayne AH, Darwent CM, Eerkens JW, Mason OK (2015). Lipid biomarkers and compound specific δ13C analysis indicate early development of a dual-economic system for the Arctic Small Tool tradition in northern Alaska. J. Archaeol. Sci..

[35] Meier-Augenstein W (2002). Stable isotope analysis of fatty acids by gas chromatography–isotope ratio mass spectrometry. Anal. Chim. Acta.

[36] Mottram HR, Dudd SN, Lawrence GJ, Stott AW, Evershed RP (1999). New chromatographic, mass spectrometric and stable isotope approaches to the classification of degraded animal fats preserved in archaeological pottery. J. Chromatogr. A.

[37] Gregg MW, Banning EB, Gibbs K, Slater GF (2009). Subsistence practices and pottery use in Neolithic Jordan: Molecular and isotopic evidence. J. Archaeol. Sci..

[38] Copley MS (2003). Direct chemical evidence for widespread dairying in prehistoric Britain. Proc. Natl. Acad. Sci..

[39] Dunne J, di Lernia S, Chłodnicki M, Kherbouche F, Evershed RP (2017). Timing and pace of dairying inception and animal husbandry practices across Holocene North Africa. Quat. Int..

[40] Dunne J (2012). First dairying in green Saharan Africa in the fifth millennium BC. Nature.

[41] Copley MS, Clark K, Evershed RP (2005). Organic-residue analysis of pottery vessels and clay balls. Changing Materialities at Çatalhoyuk: Reports from the 1995–99 Seasons.

[42] Roffet-Salque M, Lee MRF, Timpson A, Evershed RP (2017). Impact of modern cattle feeding practices on milk fatty acid stable carbon isotope compositions emphasise the need for caution in selecting reference animal tissues and products for archaeological investigations. Archaeol. Anthropol. Sci..

[43] Craig OE (2011). Ancient lipids reveal continuity in culinary practices across the transition to agriculture in Northern Europe. Proc. Natl. Acad. Sci..

[44] Salque M (2013). Earliest evidence for cheese making in the sixth millennium BC in northern Europe. Nature.

[45] Correa-Ascencio M, Robertson IG, Cabrera-Cortés O, Cabrera-Castro R, Evershed RP (2014). Pulque production from fermented agave sap as a dietary supplement in Prehispanic Mesoamerica. Proc. Natl. Acad. Sci..

[46] Kimpe K, Jacobs PA, Waelkens M (2001). Analysis of oil used in late Roman oil lamps with different mass spectrometric techniques revealed the presence of predominantly olive oil together with traces of animal fat. J. Chromatogr. A.

[47] Eerkens J (2002). The preservation and identification of Piñon resins by GC-MS in pottery from the western Great Basin. Archaeometry.

[48] Gregg MW, Brettell R, Stern B (2007). Bitumen in Neolithic Iran: Biomolecular and isotopic evidence. Archaeological Chemistry.

[49] Lucquin A, March RJ, Cassen S (2007). Analysis of adhering organic residues of two “coupes-à-socles” from the Neolithic funerary site “La Hougue Bie” in Jersey: evidences of birch bark tar utilisation. J. Archaeol. Sci..

[50] Stacey R, Cartwright C, Tanimoto S, Villing A (2010). Coatings and contents: Investigations of residues on four fragmentary sixth-century B.C. vessels from Naukratis (Egypt). Br. Museum Tech. Res. Bull..

[51] Brecoulaki H, Andreotti A, Bonaduce I, Colombini MP, Lluveras A (2012). Characterization of organic media in the wall-paintings of the “Palace of Nestor” at Pylos, Greece: Evidence for a secco painting techniques in the Bronze Age. J. Archaeol. Sci..

[52] Spades S, Russ J (2005). GC–MS analysis of lipids in prehistoric rock paints and associated oxalate coatings from the Lower Pecos Region, Texas. Archaeometry.

[53] Eckmeier E, Wiesenberg GLB (2009). Short-chain n-alkanes (C16–20) in ancient soil are useful molecular markers for prehistoric biomass burning. J. Archaeol. Sci..

[54] Chakraborty KS (2018). Enamel isotopic data from the domesticated animals at Kotada Bhadli, Gujarat, reveals specialized animal husbandry during the Indus Civilization. J. Archaeol. Sci. Rep..

[55] Chakraborty KS (2019). Subsistence-Based Economy and the Regional Interaction Processes of the Indus Civilization Borderland in Kachchh, Gujarat: A Bio-Molecular Perspective.

[56] Shirvalkar P, Rawat YS (2012). Excavation at Kotada Bhadli, District Kachchh, Gujarat: A perliminary report. Puratattva.

[57] Goyal P, Shirvalkar P, Prasad E (2020). Observations on faunal remains recovered from Kotada Bhadli. Excavation at Kotada Bhadli.

[58] Joglekar PP, Goyal P (2011). Faunal remains from Shikarpur, a Harappan site in Gujarat India. Iran. J. Archaeol. Stud..

[59] Goyal P, Joglekar PP, Kharakwal JS, Rawat YS, Osada T (2012). Archaeozoological remains from the site of Kanmer. Excavation at Kanmer (2005–2006 to 2008–2009): Kanmer archaeological research project an Indo-Japanese collaboration.

[60] Meadow RH, Patel AK, Weber S, Belcher WR (2003). Prehistoric pastoralism in northwestern South Asia from the Neolithic through the Harappan Period. Indus Ethnobiology: New perspective from the field.

[61] Patel A, Allchin B (1997). The Primary Pastoral economy of Dholavira: A first look at animals and urban life in third millennium Kutch. South Asian Archaeology 1995.

[62] Miller LJ (2004). Urban Economies in Early States: The Secondary Products Revolution in the Indus Civilization.

[63] Halstead P (1998). Mortality models and milking: Problems of uniformitarianism, optimality and equifinality reconsidered. Anthropozoologica.

[64] Gillis RE (2017). The evolution of dual meat and milk cattle husbandry in Linearbandkeramik societies. Proc. R. Soc. B Biol. Sci..

[65] Sternberg LO, Deniro MJ, Johnson HB (1984). Isotope ratios of cellulose from plants having different photosynthetic pathways. Plant Physiol..

[66] Zhang C (2012). Diets and environments of late Cenozoic mammals in the Qaidam Basin, Tibetan Plateau: Evidence from stable isotopes. Earth Planet. Sci. Lett..

[67] Cerling TE (1997). Global vegetation change through the Miocene/Pliocene boundary. Nature.

[68] Sternberg LO, Deniro MJ, Ting IP (1984). Carbon, hydrogen, and oxygen isotope ratios of cellulose from plants having intermediary photosynthetic modes. Plant Physiol..

[69] Smith BN, Epstein S (1971). Two categories of 13C/12C ratios for higher plants. Plant Physiol..

[70] Correa-Ascencio M, Evershed PR (2014). High throughput screening of organic residues in archaeological potsherds using direct acidified methanol extraction. Anal. Methods.

[71] Evershed RP, Heron C, Goad LJ (1990). Analysis of organic residues of archaeological origin by high-temperature gas chromatography and gas chromatography-mass spectrometry. Analyst.

[72] Papakosta V, Smittenberg RH, Gibbs K, Jordan P, Isaksson S (2015). Extraction and derivatization of absorbed lipid residues from very small and very old samples of ceramic potsherds for molecular analysis by gas chromatography-mass spectrometry (GC-MS) and single compound stable carbon isotope analysis by gas chromatogra. Microchem. J..

[73] Demirci Ö, Lucquin A, Craig OE, Raemaekers DCM (2020). First lipid residue analysis of Early Neolithic pottery from Swifterbant ( the Netherlands, ca 4300–4000 BC ). Archaeol. Anthropol. Sci..

[74] Carrer F (2016). Chemical analysis of pottery demonstrates prehistoric origin for high-altitude alpine dairying. PLoS ONE.

[75] Heron C (2016). First molecular and isotopic evidence of millet processing in prehistoric pottery vessels. Sci. Rep..

[76] Pecci, A. & Cau Ontiveros, M. A. *Report on the Analyses of the Organic Residues in Archaeological Samples from the Project ‘Excavating the Roman Peasant’*. *University of Barcelona* (2010).

[77] Gregg MW, Slater GF (2010). A new method for extraction, isolation and transesterification of free fatty acids from archaeological pottery. Archaeometry.

[78] Copley MS (2001). Detection of palm fruit lipids in archaeological pottery from Qasr Ibrim, Egyptian Nubia. Proc. R. Soc. B Biol. Sci..

[79] Evershed RP, Copley MS, Dickson L, Hansel FA (2008). Experimental evidence for the processing of marine animal products and other commodities containing polyunsaturated fatty acids in pottery vessels. Archaeometry.

[80] Hansel FA, Copley MS, Madureira LAS, Evershed RP (2004). Thermally produced ω-(o-alkylphenyl)alkanoic acids provide evidence for the processing of marine products in archaeological pottery vessels. Tetrahedron Lett..

[81] Meadow, R. H. Prehistoric wild sheep and sheep domestication on the eastern margin of the Middle East. in *Animal Domestication and Its Cultural Context* (eds. Crabtree, P. J., Campana, D. V. & Ryan, K.) 24–36 (University Museum, Univerity of Pennsylvania, 1989).

[82] Craig OE (2012). Distinguishing wild ruminant lipids by gas chromatography/combustion/isotope ratio mass spectrometry. Rapid Commun. Mass Spectrom..

[83] Pokharia, A. K. Floral Remains. in *Excavation at Kanmer (2005–06 to 2008–09): Kanmer archaeological research project an Indo-Japanese collaboration* (eds. Kharakwal, J. S., Rawat, Y. S. & Osada, T.) 795–812 (Indus project, research institute for humanity and Nature, 2012).

[84] Steele VJ, Stern B, Stott AW (2010). Olive oil or lard?: distinguishing plant oils from animal fats in the archeological record of the eastern Mediterranean using gas chromatography/combustion/isotope ratio mass spectrometry. Rapid Commun. Mass Spectrom..

[85] Spangenberg JE, Ogrinc N (2001). Authentication of vegetable oils by bulk and molecular carbon isotope analyses with emphasis on olive oil and pumpkin seed oil. J. Agric. Food Chem..

[86] Hammann S, Cramp LJE (2018). Towards the detection of dietary cereal processing through absorbed lipid biomarkers in archaeological pottery. J. Archaeol. Sci..

[87] Colonese AC (2017). New criteria for the molecular identification of cereal grains associated with archaeological artefacts. Sci. Rep..

[88] Courel B (2020). Organic residue analysis shows sub-regional patterns in the use of pottery by Northern European hunter-gatherers. R. Soc. Open Sci..

[89] Hendy J (2018). Ancient proteins from ceramic vessels at Çatalhöyük West reveal the hidden cuisine of early farmers. Nat. Commun..

[90] Chase B, Meiggs D, Ajithprasad P (2020). Pastoralism, climate change, and the transformation of the Indus Civilization in Gujarat: Faunal analyses and biogenic isotopes. J. Anthropol. Archaeol..

[91] Margabandhu C, Agarwal DP, Ghosh A (1973). Technology of trasport vehicles in early India. Radiocarbon and Indian Archaeology.

[92] Fairservis WJ (1986). Cattle. Exped. Mag..

[93] Chase B, Frenez D, Jamison GM, Law RW, Vidale M, Meadow RH (2018). Family matters in Gujarat. Walking with the Unicorn: Social Organization and Material Culture in Ancient South Asia.

