# Supplementary material for: Compound specific isotope analysis of lipid residues provides the earliest direct evidence of dairy product processing in South Asia
Source: Sci Rep. 2020 Sep 30;10:16095. doi: 10.1038/s41598-020-72963-y (PMC7528006; doi:10.1038/s41598-020-72963-y)
Supplement: Supplementary file 1 — Supplementary file1 [file 41598_2020_72963_MOESM1_ESM.docx]

**Compound specific isotope analysis of lipid residues provides the earliest direct evidence of dairy product processing in South Asia.**

**Authors: Kalyan Sekhar Chakraborty^1,*^, Greg F. Slater^2^, Heather M.-L. Miller^1^, Prabodh Shirvalkar^3^, Yadubirsingh Rawat^4^.**

**Affiliation:**

**^1^** Department of Anthropology, University of Toronto, Mississauga, Ontario, Canada.

**^2^** School of Geography and Earth Sciences, McMaster University, Hamilton, Ontario, Canada.

**^3^** Department of A.I.H.C. and Archaeology, Deccan College Postgraduate and Research Institute, Pune, Maharashtra, India.

**^4^** State Department of Archaeology and Museum, Gandhinagar, Gujarat, India.

| **Sample ID** | **Shape** | **Fabric** | **Surface treatment** | **Portion of vessel** | **Molecular composition (one step methanolic extraction)** | **Extraction method** | **Quantity per sample (excluding phthalic acid) using one step methanolic extraction­** | **Fatty acids yields: μg/g** | | **Tentative identification of source based on C16:0/C18:0** | **GC-IRMS values (δ13C)‰** | | **Δ13c (C18:0-C16:0)‰** | **Source based on isotopic composition** |
| --- | --- | --- | --- | --- | --- | --- | --- | --- | --- | --- | --- | --- | --- | --- |
|  |  |  |  |  |  |  |  | **C16:0** | **C18:0** |  | **C16:0** | **C18:0** |  |  |
| **KB-5** | Cooking vessel | Coarse red-ware | Untreated | Body | Saturated fatty acids: C14:0, C15:0, C16:0, C17:0, C18:0, C20:0, C21:0, C22:0, C23:0, C24:0  Unsaturated fatty acids: C16:1, C18:1  Branched fatty acids: C15br, C17br  Others: Phthalic acid | One step acidified methanolic extraction | 59μg/g | 23 | 24 | Animal fat | -14 | -19.4 | -5.4 | Ruminant dairy |
| **KB-10** | Cooking vessel | Coarse red-ware | Untreated | Body | Saturated fatty acids: C14:0, C15:0, C16:0, C17:0, C18:0, C20:0  Unsaturated fatty acids: C16:1, C18:1  Branched fatty acids: C15br  Others: Phthalic acid | One step acidified methanolic extraction | 10μg/g | 5 | 2 | Plant oil | - | - | - | - |
| **KB-17** | Pot/Jar | Grey-ware | Untreated | Body | Saturated fatty acids: C14:0, C15:0, C16:0, C17:0, C18:0, C19:0, C20:0, C21:0, C22:0, C23:0, C24:0, C25:0, C26:0  Unsaturated fatty acids: C16:1, C18:1  Branched fatty acids: C15br, C17br  Others: Phthalic acid | One step acidified methanolic extraction | 16μg/g | 7 | 6 | Animal fat | - | - |  | - |
| **KB-19** | Cooking vessel | Coarse red-ware | Untreated | Body | Saturated fatty acids: C14:0, C15:0, C16:0, C17:0, C18:0, C20:0  Unsaturated fatty acids: C16:1, C18:1  Branched fatty acids: C15br  Others: Phthalic acid | One step acidified methanolic extraction | 14μg/g | 5 | 3 | Animal fat | - | - |  | - |
| **KB-22** | Bowl | Coarse red-ware | Untreated | body | Saturated fatty acids: C12:0, C13:0, C14:0, C15:0, C16:0, C17:0, C18:0, C19:0, C20:0, C21:0, C22:0, C23:0, C24:0, C25:0, C26:0  Unsaturated fatty acids: C16:1, C18:1  Branched fatty acids: C15br, C16br, C17br, C18br  Dicarboxylic acid: Octanedoic acid, Nonanedioic acid, Decanedoic acid, Undecanedoic acid  Others: Phthalic acid | Conventional solvent extraction, BSTFA derivatization,  and  one step acidified methanolic extraction | 262μg/g | 83 | 49 | Animal fat | -15 | -20 | -5 | Ruminant dairy |
| **KB-23** | Cooking vessel | Coarse red-ware | Untreated | Body | Saturated fatty acids: C14:0, C15:0, C16:0, C17:0, C18:0, C20:0  Unsaturated fatty acids: C18:1  Others: Phthalic acid | One step acidified methanolic extraction | 8μg/g | 3 | 3 | Animal fat | - | - |  | - |
| **KB-27** | Cooking vessel | Coarse red-ware | Untreated | Body | Saturated fatty acids: C14:0, C15:0, C16:0, C17:0, C18:0, C20:0  Unsaturated fatty acids: C16:1, C18:1  Branched fatty acids: C15br, C17br  Others: Phthalic acid | One step acidified methanolic extraction | 28μg/g | 12 | 15 | Animal fat | -29.7 | -30.5 | -0.8 | Mixed non-ruminant and ruminant adipose |
| **KB-30** | Cooking vessel | Coarse red-ware | Untreated | Body | Saturated fatty acids: C14:0, C15:0, C16:0, C17:0, C18:0, C20:0  Unsaturated fatty acids: C16:1, C18:1  Others: Phthalic acid | One step acidified methanolic extraction | 31μg/g | 9 | 11 | Animal fat | -24.5 | -21.6 | 2.9 | Non-ruminant adipose |
| **KB-31** | Cooking vessel | Coarse red-ware | Untreated | Body | Saturated fatty acids: C14:0, C15:0, C16:0, C17:0, C18:0, C19:0, C20:0, C21:0, C22:0, C23:0 C24:0  Unsaturated fatty acids: C16:1, C18:1  Branched fatty acids: C15br, C17br  Others: Phthalic acid | One step acidified methanolic extraction | 29μg/g | 9 | 12 | Aniimal fat | -16.3 | -17.1 | -0.8 | Mixed non-ruminant and ruminant adipose |
| **KB-35** | Cooking vessel | Coarse red-ware | Untreated | Body | Saturated fatty acids: C14:0, C15:0, C16:0, C17:0, C18:0, C20:0  Others: Phthalic acid | One step acidified methanolic extraction | 12μg/g | 4 | 7 | Animal fat | - | - |  | - |
| **KB-38** | Cooking vessel | Coarse red-ware | Untreated | Body | Saturated fatty acids: C14:0, C15:0, C16:0, C17:0, C18:0, C20:0  Unsaturated fatty acids: C18:1  Branched fatty acids: C15br  Others: Phthalic acid | One step acidified methanolic extraction | 7μg/g | 3 | 3 | Animal fat | - | - |  | - |
| **KB-39** | Cooking vessel | Coarse red-ware | Untreated | Body | Saturated fatty acids: C14:0, C15:0, C16:0, C17:0, C18:0, C20:0  Unsaturated fatty acids: C18:1  Others: Phthalic acid | Conventional solvent extraction, BSTFA derivatization,  and  one step acidified methanolic extraction | 10μg/g | 4 | 3 | Animal fat | - | - |  | - |
| **KB-42** | Cooking vessel | Coarse red-ware | Untreated | Body | Saturated fatty acids: C14:0, C15:0, C16:0, C17:0, C18:0, C20:0  Unsaturated fatty acids: C16:1, C18:1  Branched fatty acids: C15br  Others: Phthalic acid | One step acidified methanolic extraction | 14μg/g | 7 | 6 | Animal fat | - | - |  | - |
| **KB-48** | Cooking vessel | Coarse red-ware | Untreated | Body | Saturated fatty acids: C14:0, C15:0, C16:0, C17:0, C18:0, C20:0  Unsaturated fatty acids: C16:1, C18:1  Branched fatty acids: C15br, C17br  Others: Phthalic acid | One step acidified methanolic extraction | 22μg/g | 9 | 9 | Animal fat | -29.7 | -30.1 | -0.4 | Mixed non-ruminant and ruminant adipose |
| **KB-52** | Cooking vessel | Coarse red-ware | Untreated | Body | Saturated fatty acids: C12:0, C14:0, C15:0, C16:0, C17:0, C18:0, C20:0  Unsaturated fatty acids: C16:1, C18:1  Branched fatty acids: C15br, C17br  Others: Phthalic acid | One step acidified methanolic extraction | 30μg/g | 10 | 13 | Animal fat | -28.2 | -28.8 | -0.6 | Mixed non-ruminant and ruminant adipose pose |
| **KB-59** | Cooking vessel | Coarse red-ware | Untreated | Body | Saturated fatty acids: C14:0, C15:0, C16:0, C17:0, C18:0, C20:0  Unsaturated fatty acids: C16:1, C18:1  Others: Phthalic acid | One step acidified methanolic extraction | 10μg/g | 3 | 2 | Animal fat | - | - |  | - |
| **KB-60** | Cooking vessel | Coarse red-ware | Untreated | Body | Saturated fatty acids: C12:0, C14:0, C15:0, C16:0, C17:0, C18:0, C19:0, C20:0, C21:0, C22:0, C23:0, C24:0  Unsaturated fatty acids: C16:1, C18:1  Branched fatty acids: C15br, C16br, C17br  Others: 4,8,12 TMTD, Phthalic acid | One step acidified methanolic extraction | 38μg/g | 17 | 10 | Animal fat | -16.7 | -23.3 | -6.5 | Ruminant dairy |
| **KB-62** | Cooking vessel | Coarse red-ware | Untreated | Body | Saturated fatty acids: C14:0, C16:0, C18:0, C20:0  Unsaturated fatty acids: C18:1  Others: Phthalic acid | One step acidified methanolic extraction | 18μg/g | 7 | 8 | Animal fat | - | - |  | - |
| **KB-63** | Cooking vessel | Coarse red-ware | Untreated | Body | Saturated fatty acids: C14:0, C15:0, C16:0, C17:0, C18:0, C20:0  Unsaturated fatty acids: C18:1  Others: Phthalic acid | One step acidified methanolic extraction | 10μg/g | 3 | 4 | Animal fat |  |  |  |  |
| **KB-65** | Cooking vessel | Coarse red-ware | Untreated | Body | Saturated fatty acids: C14:0, C15:0, C16:0, C17:0, C18:0, C20:0, C22:0, C24:0  Unsaturated fatty acids: C16:1, C18:1  Others: Phthalic acid | One step acidified methanolic extraction | 19μg/g | 8 | 2 | Plant oil | - | - |  | - |
| **KB-69** | Bowl | Coarse red-ware | Red slip | Rim | Saturated fatty acids: C12:0, C13:0, C14:0, C15:0, C16:0, C17:0, C18:0, C20:0  Unsaturated fatty acids: C16:1, C18:1  Branched fatty acids: C15br and C17br  Dicarboxylic acid: Nonanedioic acid  Others: Phthalic acid | One step acidified methanolic extraction | 39μg/g | 16 | 10 | Animal fat | -21.4 | -27.1 | -5.7 | Ruminant dairy |
| **KB-70** | Bowl | Grey-ware | Untreated (Grooved mark on the rim) | Rim | Saturated fatty acids: C12:0, C13:0, C14:0, C15:0, C16:0, C17:0, C18:0  Unsaturated fatty acids: C16:1, C18:1  Branched fatty acids: C15br and C17br  Dicarboxylic acid: Octanedioic acid, Nonanedioic acid  Others: Phthalic acid | One step acidified methanolic extraction | 24μg/g | 7 | 5 | Animal fat | - | - |  | - |
| **KB-71** | Bowl | Red-ware | Red slip | Body | Saturated fatty acids: C14:0, C15:0, C16:0, C17:0, C18:0, C19:0, C20:0, C22:0, C24:0  Unsaturated fatty acids: C16:1, C18:1  Branched fatty acids: C15br, C16br and C17br  Others: Phthalic acid | One step acidified methanolic extraction | 14μg/g | 5 | 2 | Animal fat | - | - |  | - |
| **KB-75** | Bowl | Red-ware | Untreated | Body | Saturated fatty acids: C14:0, C15:0, C16:0, C17:0, C18:0  Unsaturated fatty acids: C16:1, C18:1  Branched fatty acids: C15br, C17br  Others: Phthalic acid | One step acidified methanolic extraction | 11μg/g | 6 | 4 | Animal fat | - | - |  | - |
| **KB-77** | Bowl | Red-ware | Red slip | Body | Saturated fatty acids: C14:0, C15:0, C16:0, C17:0, C18:0, C20:0  Unsaturated fatty acids: C16:1, C18:1  Others: Phthalic acid | One step acidified methanolic extraction | 9μg/g | 4 | 4 | Animal fat | - | - |  | - |
| **KB-78** | Bowl | Coarse red-ware | Untreated | Rim | Saturated fatty acids: C12:0, C14:0, C15:0, C16:0, C17:0, C18:0, C20:0  Unsaturated fatty acids: C16:1, C18:1  Others: Phthalic acid | One step acidified methanolic extraction | 34μg/g | 5 | 3 | Animal fat | - | - |  | - |
| **KB-84** | Carinated bowl | Redware | Untreated | Rim | Saturated fatty acids: C14:0, C15:0, C16:0, C17:0, C18:0, C19:0, C20:0, C22:0, C24:0  Unsaturated fatty acids: C16:1, C18:1  Branched fatty acids: C15br, C16br, C17br  Dicarboxylic acid: Decanedioic acid  Others: Phthalic acid | One step acidified methanolic extraction | 36μg/g | 14 | 10 | Animal fat | -17.9 | -22 | -4.1 | Ruminant dairy/deer |
| **KB-105** | Bowl | Red-ware | Red slip and painted | Rim | Saturated fatty acids: C14:0, C15:0, C16:0, C17:0, C18:0, C20:0, C22:0  Unsaturated fatty acids: C16:1, C18:1  Branched fatty acids: C15br, C16br, and C17br  Dicarboxylic acid: Nonanedioic acid  Others: Phthalic acid | One step acidified methanolic extraction | 10μg/g | 3 | 4 | Animal fat | - | - |  | - |
| **KB-106** | Carinated bowl | Red-ware | Untreated | Rim | Saturated fatty acids: C12:0, C13:0, C14:0, C15:0, C16:0, C17:0, C18:0, C19:0, C20:0, C21:0, C22:0, C24:0, C26:0  Unsaturated fatty acids: C16:1, C18:1, C20:1, C22:1  Branched fatty acids: C15br, C16br, and C17br  Dicarboxylic acid: Nonanedioic acid, Decanedioic acid, Undecanedioic acid  Others: Phthalic acid, 4,8,12 TMTD, Phytanic acid, APAAs (18,20,22) | Conventional solvent extraction, BSTFA derivatization,  and  one step acidified methanolic extraction | 91μg/g | 34 | 21 | Aquatic fat | - | - | - | - |
| **KB-107** | Pot | Red-ware | Red slip | Rim | Saturated fatty acids: C14:0, C15:0, C16:0, C17:0, C18:0, C19:0, C20:0, C22:0, C24:0  Unsaturated fatty acids: C16:1, C18:1  Branched fatty acids: C15br, C17br  Others: Phthalic acid | One step acidified methanolic extraction | 18μg/g | 8 | 6 | Animal fat | - | - |  | - |
| **KB-108** | Bowl | Red-ware | Red slip, painted | Body | Saturated fatty acids: C12:0, C13:0, C14:0, C15:0, C16:0, C17:0, C18:0, C19:0, C20:0, C22:0  Unsaturated fatty acids: C16:1, C18:1  Branched fatty acids: C15br and C17br  Dicarboxylic acid: Nonanedioic acid, Decanedioic acid, Undecanedioic acid  Others: Phthalic acid | One step acidified methanolic extraction | 18μg/g | 7 | 6 | Animal fat | - | - |  | - |
| **KB-109** | Bowl | Red-ware | Untreated | Body | Saturated fatty acids: C12:0, C13:0, C14:0, C15:0, C16:0, C17:0, C18:0, C19:0, C20:0, C21:0, C22:0, C23:0 C24:0, C25:0 C26:0, C28:0  Unsaturated fatty acids: C16:1, C18:1, C18:2  Branched fatty acids: C15br, C16br, and C17br  Dicarboxylic acid: Nonanedioic acid  Others: Phthalic acid | Conventional solvent extraction, BSTFA derivatization,  and  one step acidified methanolic extraction | 45μg/g | 15 | 7 | Animal fat | -19.9 | -21.8 | -1.9 | Ruminant adipose |
| **KB-110** | Bowl | Red-ware | Untreated | Body | Saturated fatty acids: C12:0, C13:0, C14:0, C15:0, C16:0, C17:0, C18:0, C19:0, C20:0  Unsaturated fatty acids: C16:1, C18:1  Branched fatty acids: C15br, C16br, and C17br  Dicarboxylic acid: Nonanedioic acid  Others: Phthalic acid | One step acidified methanolic extraction | 15μg/g | 6 | 3 | Animal fat | - | - |  | - |
| **KB-115** | Hole-mouthed lid | Red-ware | Red slip | Fragment of the lid | Saturated fatty acids: C12:0, C13:0, C14:0, C15:0, C16:0, C17:0, C18:0, C19:0, C20:0, C21:0, C22:0, C23:0, C24:0  Unsaturated fatty acids: C16:1, C18:1,  Branched fatty acids: C15br, C17br  Dicarboxylic acid: Nonanedioic acid, Decanedioic acid, Undecanedioic acid  Others: Phthalic acid, Phytanic acid | One step acidified methanolic extraction | 85μg/g | 28 | 17 | Animal fat | -25.9 | -26.5 | -0.6 |  |
| **KB-117** | Bowl | Grey-ware | Black slip | Rim | Saturated fatty acids: C12:0, C13:0, C14:0, C15:0, C16:0, C17:0, C18:0, C19:0, C20:0  Unsaturated fatty acids: C16:1, C18:1  Branched fatty acids: C15br, C16br, and C17br  Others: Phthalic acid | One step acidified methanolic extraction | 7μg/g | 3 | 3 | Animal fat | - | - |  | - |
| **KB-125** | Carinated bowl | Red-ware | Red slip | Body | Saturated fatty acids: C12:0, C14:0, C15:0, C16:0, C17:0, C18:0, C20:0  Unsaturated fatty acids: C16:1, C18:1  Branched fatty acids: C15br, C16br, and C17br  Others: Phthalic acid | One step acidified methanolic extraction | 14μg/g | 7 | 4 | Animal fat | - | - |  | - |
| **KB-135** | Perforated vessel | Red-ware | Untreated | Fragment of the body | Saturated fatty acids: C12:0, C14:0, C15:0, C16:0, C17:0, C18:0, C19:0, C20:0, C21:0, C22:0, C23:0, C24:0, C25:0, C26:0  Unsaturated fatty acids: C16:1, C18:1,  Branched fatty acids: C15br, C17br  Dicarboxylic acid: Nonanedioic acid  Others: Phthalic acid | One step acidified methanolic extraction | 48μg/g | 26 | 12 | Animal fat | -21 | -24.3 | -3.3 | Mixed ruminant adipose and dairy/deer |
| **KB-136** | Pot/Jar | Coarse red-ware | Untreated | Body | Saturated fatty acids: C14:0, C15:0, C16:0, C17:0, C18:0, C20:0  Unsaturated fatty acids: C16:1, C18:1  Branched fatty acids: C15br, C17br  Others: Phthalic acid | One step acidified methanolic extraction | 18μg/g | 9 | 2 | Plant oil | - | - |  | - |
| **KB-143** | Pot/Jar | Red-ware | Untreated | Body | Saturated fatty acids: C12:0, C13:0, C14:0, C15:0, C16:0, C17:0, C18:0,  Unsaturated fatty acids: C16:1, C18:1, C18:2  Branched fatty acids: C15br, C17br  Dicarboxylic acids: Nonanedoic acid  Others: Phthalic acid | Conventional solvent extraction, BSTFA derivatization,  and  one step acidified methanolic extraction | 14μg/g | 6 | 2 | Plant oil | - | - |  | - |
| **KB-154** | Cooking vessel | Coarse red-ware | Untreated | Body | Saturated fatty acids: C14:0, C15:0, C16:0, C17:0, C18:0, C20:0  Unsaturated fatty acids: C16:1, C18:1  Branched fatty acids: C15br  Others: Phthalic acid | One step acidified methanolic extraction | 11μg/g | 5 | 2 | Plant oil | - | - |  | - |
| **KB-156** | Bowl | Red-ware | Untreated | Rim | Saturated fatty acids: C12:0, C14:0, C15:0, C16:0, C17:0, C18:0, C20:0  Unsaturated fatty acids: C16:1, C18:1  Branched fatty acids: C15br, C16br, and C17br  Dicarboxylic acid: Nonanedioic acid  Others: Phytanic acid, and Phthalic acid | One step acidified methanolic extraction | 21μg/g | 12 | 4 | Animal fat | -25.1 | -25.6 | -0.5 | Mixed non-ruminant and ruminant adipose |
| **KB-157** | Pot/Jar | Red-ware | Red slip | Body | Saturated fatty acids: C14:0, C15:0, C16:0, C17:0, C18:0, C20:0  Unsaturated fatty acids: C16:1, C18:1  Branched fatty acids: C15br, C17br  Others: Phthalic acid | One step acidified methanolic extraction | 10μg/g | 5 | 5 | Animal fat | - | - |  | - |
| **KB-159** | Bowl | Coarse red-ware | Black slip | Rim | Saturated fatty acids: C12:0, C14:0, C15:0, C16:0, C17:0, C18:0, C20:0  Unsaturated fatty acids: C16:1, C18:1, C18:2  Branched fatty acids: C15br, C16br, and C17br  Dicarboxylic acid: Nonanedioic acid, Decanedioic acid, Undecanedioic acid  Others: Phytanic acid, and Phthalic acid | One step acidified methanolic extraction | 77μg/g | 36 | 24 | Animal fat | -17.6 | -23.1 | -5.5 | Ruminant dairy |
| **KB-167** | Large deep bowl/basin | Red-ware | Untreated | Rim | Saturated fatty acids: C14:0, C15:0, C16:0, C17:0, C18:0, C20:0  Unsaturated fatty acids: C16:1, C18:1  Branched fatty acids: C17br  Dicarboxylic acid: Nonanedioic acid, Decanedioic acid, Undecanedioic acid  Others: Phytanic acid, and Phthalic acid | One step acidified methanolic extraction | 45μg/g | 24 | 14 | Animal fat | -25.6 | -20.7 | 4.9 | Non-ruminant adipose |
| **KB-168** | Cooking vessel | Coarse red-ware | Untreated | Body | Saturated fatty acids: C14:0, C15:0, C16:0, C17:0, C18:0, C20:0  Unsaturated fatty acids: C16:1, C18:1  Others: Phthalic acid | One step acidified methanolic extraction | 16μg/g | 8 | 6 | Animal fat | - | - |  | - |
| **KB-171** | Bowl | Coarse red-ware | Red slip | body | Saturated fatty acids: C14:0, C15:0, C16:0, C17:0, C18:0, C19:0, C20:0  Unsaturated fatty acids: C16:1, C18:1  Branched fatty acids: C15br, C16br, and C17br  Dicarboxylic acid: Undecanedioic acid  Others: Phytanic acid, Phthalic acid | One step acidified methanolic extraction | 105μg/g | 38 | 43 | Animal fat | -14.5 | -16.3 | -1.8 | Ruminant adipose |
| **KB-172** | Cooking vessel | Coarse red-ware | Untreated | Neck | Saturated fatty acids: C14:0, C15:0, C16:0, C17:0, C18:0, C20:0  Unsaturated fatty acids: C16:1, C18:1  Branched fatty acids: C15br, C17br  Others: Phthalic acid | One step acidified methanolic extraction | 12μg/g | 4 | 5 | Animal fat | - | - |  | - |
| **KB-173** | Channel-handled hemispherical bowl/ladle | Red-ware | Red slip | Body with part of handle | Saturated fatty acids: C12:0, C13:0, C14:0, C15:0, C16:0, C17:0, C18:0, C19:0, C20:0  Unsaturated fatty acids: C16:1, C18:1  Branched fatty acids: C15br, C17br  Others: Phthalic acid | One step acidified methanolic extraction | 20μg/g | 11 | 5 | Animal fat | -21.1 | -23.5 | -2.4 | Ruminant adipose |
| **KB-182** | Bowl | Red-ware | Untreated | Body | Saturated fatty acids: C14:0, C15:0, C16:0, C17:0, C18:0, C19:0, C20:0, C22:0  Unsaturated fatty acids: C16:1, C18:1, C18:2  Dicarboxylic acid: Decanedoic acid, Undecanedioic acid  Others: Phthalic acid | One step acidified methanolic extraction | 24μg/g | 12 | 8 | Animal fat | - | - |  | - |
| **KB-185** | Pot/Jar | Red-ware | Untreated | Body | Saturated fatty acids: C12:0, C13:0, C14:0, C15:0, C16:0, C17:0, C18:0, C19:0, C20:0, C21:0, C22:0  Unsaturated fatty acids: C16:1, C18:1  Branched fatty acids: C15br, C17br  Dicarboxylic acids: Nonanedoic acid, Decanedoic acid, Undecanedoic acid  Others: Phthalic acid and Phytanic acid | One step acidified methanolic extraction | 11μg/g | 6 | 3 | Animal fat | - | - |  | - |
| **KB-189** | Cooking vessel | Coarse red-ware | Untreated | Body | Saturated fatty acids: C16:0, C17:0, C18:0, C20:0  Unsaturated fatty acids: C16:1, C18:1  Others: Phthalic acid | One step acidified methanolic extraction | 6μg/g | 2 | 3 | Animal fat | - | - |  | - |
| **KB-191** | Cooking vessel | Coarse red-ware | Untreated | Body | Saturated fatty acids: C14:0, C15:0, C16:0, C17:0, C18:0, C20:0  Unsaturated fatty acids: C16:1, C18:1  Others: Phthalic acid | One step acidified methanolic extraction | 50μg/g | 22 | 25 | Animal fat | -29.4 | -28.8 | 0.6 | Mixed non-ruminant and ruminant adipose |
| **KB-199** | Globular pot | Coarse red-ware | Untreated | Rim | Saturated fatty acids: C14:0, C15:0, C16:0, C17:0, C18:0, C20:0  Unsaturated fatty acids: C18:1  Branched fatty acids: C17br  Others: Phthalic acid | One step acidified methanolic extraction | 15μg/g | 8 | 5 | Animal fat | - | - |  | - |
| **KB-202** | Cooking vessel | Coarse red-ware | Untreated | Body | Saturated fatty acids: C12:0, C14:0, C15:0, C16:0, C17:0, C18:0  Unsaturated fatty acids: C16:1, C18:1  Branched fatty acids: C15br  Others: Phthalic acid | One step acidified methanolic extraction | 36μg/g | 8 | 2 | Plant oil | - | - |  | - |
| **KB-207** | Bowl | Grey-ware | Black slip | Rim | Saturated fatty acids: C12:0, C14:0, C16:0, C17:0, C18:0,  Others: Phthalic acid | One step acidified methanolic extraction | 9μg/g | 3 | 2 | Animal fat | - | - |  | - |
| **KB-213** | Bowl | Kaolinite-ware | Red slip | Rim | Saturated fatty acids: C15:0, C16:0, C17:0, C18:0, C20:0  Unsaturated fatty acids: C16:1, C18:1  Branched fatty acids: C17br  Others: Phthalic acid | One step acidified methanolic extraction | 5μg/g | 2 | 2 | Animal fat | - | - |  | - |
| **KB-215** | Bowl | Red-ware | Red slip | Rim | Saturated fatty acids: C12:0, C14:0, C15:0, C16:0, C17:0, C18:0, C20:0  Unsaturated fatty acids: C16:1, C18:1  Branched fatty acids: C15br, C16br, and C17br  Dicarboxylic acid: Nonanedioic acid  Others: Phthalic acid | One step acidified methanolic extraction | 17μg/g | 9 | 4 | Animal fat | -26.4 | -27.9 | -1.5 | Mixed non-ruminant and ruminant adipose |
| **KB-225** | Channel-handled hemispherical bowl/ladle | Red-ware | Red slip | Body with part of handle | Saturated fatty acids: C12:0, C13:0, C14:0, C15:0, C16:0, C17:0, C18:0, C19:0, C20:0  Unsaturated fatty acids: C16:1, C18:1  Branched fatty acids: C15br, C17br  Others: Phthalic acid | One step acidified methanolic extraction | 22μg/g | 10 | 7 | Animal fat | -22.1 | -25.2 | -3.1 | Mixed ruminant adipose and dairy/deer |
| **A-110** | Channel-handled hemispherical bowl/ladle | Red-ware | Red slip | Mostly the handle | Saturated fatty acids: C12:0, C13:0, C14:0, C15:0, C16:0, C17:0, C18:0, C19:0, C20:0  Unsaturated fatty acids: C16:1, C18:1  Branched fatty acids: C15br, C17br  Others: Phthalic acid | One step acidified methanolic extraction | 22μg/g | 7 | 3 | Animal fat | - | - |  | - |

**Supplementary Information table S1**: Details of pottery samples from Kotada Bhadli used for organic residue analysis.

| Sample id | Type of vessels | δ^13^C value of C_16:0_ (‰) | δ^13^C value of C_18:0_ (‰) | Δ^13^C (C_18:0_-C_16:0_ (‰) | Probable source of lipids |
| --- | --- | --- | --- | --- | --- |
| KB-60 | Coarse red-ware cooking vessels | -16.7 | -23.3 | -6.5 | Ruminant dairy |
| KB-106 | Plain red-ware carinated bowl | -19.1 | -24.9 | -5.8 | Ruminant dairy |
| KB-69 | Red-sliped coarse red-ware bowl | -21.4 | -27.1 | -5.7 | Ruminant dairy |
| KB-159 | Plain coarse red-ware bowl | -17.6 | -23.1 | -5.5 | Ruminant dairy |
| KB-5 | Coarse red-ware cooking vessels | -14 | -19.4 | -5.4 | Ruminant dairy |
| KB-22 | Plain coarse red-ware bowl | -15 | -20 | -5 | Ruminant dairy |
| KB-84 | Red-ware carinated bowl | -17.9 | -22 | -4.1 | Ruminant dairy/deer |
| KB-135 | Perforated jar | -21 | -24.3 | -3.3 | Mixed ruminant adipose and dairy/deer |
| KB-225 | Channel-handled spherical bowl/ladle | -22.1 | -25.2 | -3.1 | Mixed ruminant adipose and dairy/deer |
| KB-173 | Channel-handled hemi-spherical bowl/ladle | -21.1 | -23.5 | -2.4 | Ruminant adipose |
| KB-109 | Plain red-ware bowl | -19.9 | -21.8 | -1.9 | Ruminant adipose |
| KB-171 | Red-slipped coarse red-ware bowl | -14.5 | -16.3 | -1.8 | Ruminant adipose |
| KB-215 | Red-slipped red-ware bowl | -26.4 | -27.9 | -1.5 | Mixed ruminant adipose |
| KB-31 | Coarse red-ware cooking vessels | -16.3 | -17.1 | -0.8 | Ruminant adipose |
| KB-27 | Coarse red-ware cooking vessels | -29.7 | -30.5 | -0.8 | Ruminant adipose |
| KB-52 | Coarse red-ware cooking vessels | -28.2 | -28.8 | -0.6 | Mixed non-ruminant and ruminant adipose |
| KB-115 | Hole-mouthed lid of bottle | -25.9 | -26.5 | -0.6 | Mixed non-ruminant and ruminant adipose |
| KB-156 | Plain red-ware bowl | -25.1 | -25.6 | -0.5 | Mixed non-ruminant and ruminant adipose |
| KB-48 | Coarse red-ware cooking vessels | -29.7 | -30.1 | -0.4 | Mixed non-ruminant and ruminant adipose |
| KB-191 | Coarse red-ware cooking vessels | -29.4 | -28.8 | 0.6 | Mixed non-ruminant and ruminant adipose |
| KB-30 | Coarse red-ware cooking vessels | -24.5 | -21.6 | 2.9 | Non-ruminant adipose |
| KB167 | Plain red-ware large deep bowl | -25.6 | -20.7 | 4.9 | Non-ruminant adipose |

Supplementary Information table S2: GC-IRMS results of pottery samples from Kotada Bhadli, sorted based on the source of animal fats.

**Method**

**Conventional Chloroform-Methanol Extraction and BSTFA Derivatization**

The conventional chloroform-methanol extraction method was used for 5 samples. For each sample, approximately 2 gm of ceramic powder was weighed and transferred into a 20 ml glass vial and 10 ml of 2:1 v/v chloroform-methanol was added and sonicated for 20 min (2X) to obtain a total lipid extract (TLE). The total lipid extracts (TLE) were then centrifuged at 2500 rpm for 15 min and the supernatant liquid containing the TLE was extracted, leaving behind the powdered sherds. The total lipid extract solvent was then evaporated to dryness under a gentle stream of nitrogen. The TLE was then derivatized with BSTFA+1%TMCS at 70˚C for one hour. 20 μl of *n-*triacontane (1 mg/ml) was added as an internal standard, and the solution was then diluted to 300 μl, and was analyzed by a GC-MS.

The analysis was carried out at McMaster University, Ontario, Canada, on an Agilent 6890 GC equipped with a 5973 quadrupole mass spectrophotometer. The column used was an Agilent DB5-MS + DG, 30 m×0.25 mm with a 0.25 μm thickness. Helium was the carrier gas. The initial temperature of GC was set at 50˚C held for two minutes, ramped to 300˚C at a rate of 10˚C/min, and was held at the final temperature for ten minutes. 1ul of sample was introduced to the GC by splitless injection. The MS was operated in a scan mode with 12 minutes of sample delay, the MS quad temperature was set at 150˚C and the MS source temperature was set at 230˚C. The data acquisition was between *m/z* 50 and 450. Acquisition and data analysis were performed using ChemStation D.01.02 software.
