# Supplementary figures and images for: Compound specific isotope analysis of lipid residues provides the earliest direct evidence of dairy product processing in South Asia
Source: Sci Rep. 2020 Sep 30;10:16095. doi: 10.1038/s41598-020-72963-y (PMC7528006; doi:10.1038/s41598-020-72963-y)

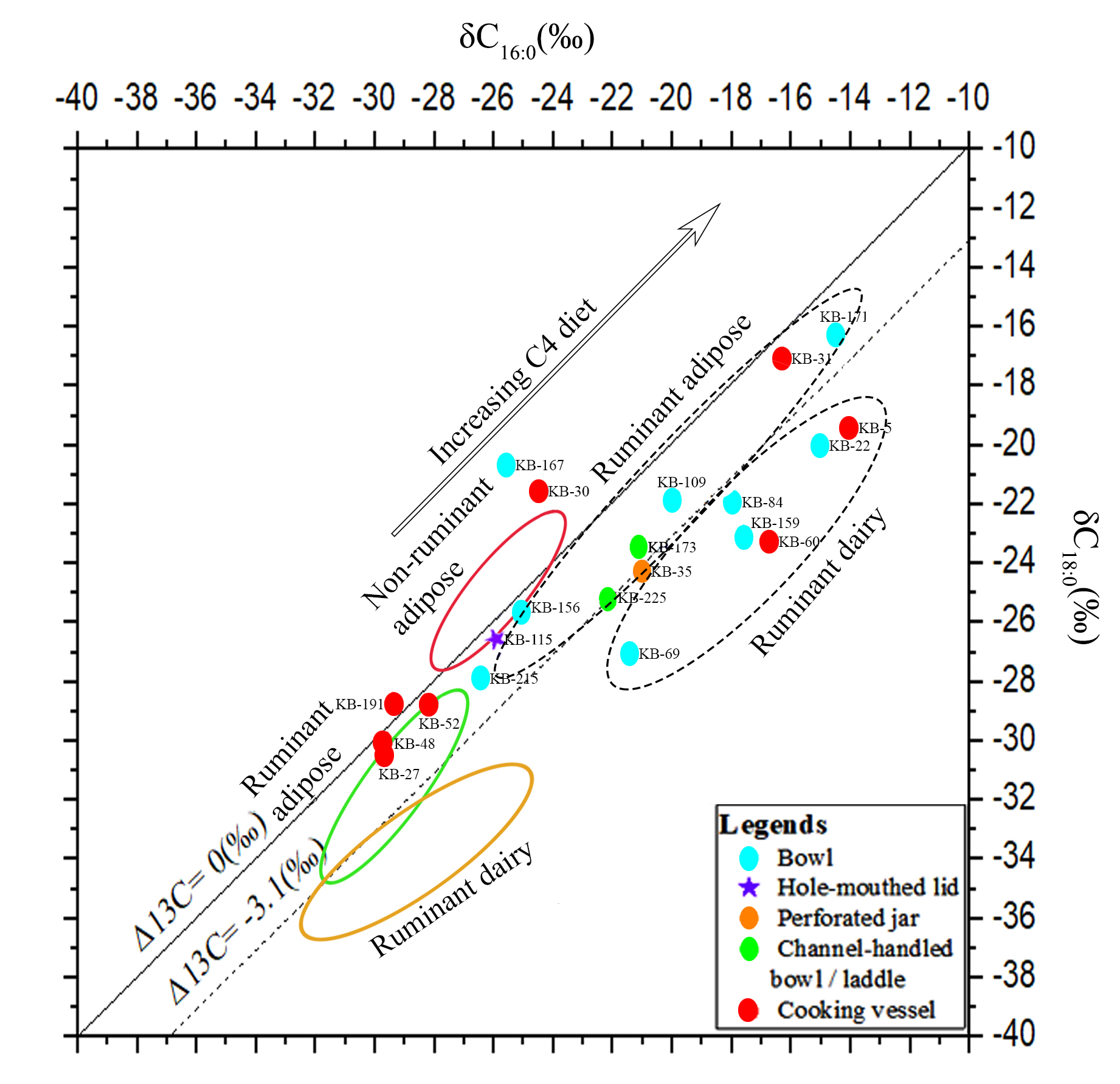

Supplement: Supplementary file 2 — Supplementary file2 [file 41598_2020_72963_MOESM2_ESM.jpg]
